# Molecular and Cellular Mechanisms Affected in ALS

**DOI:** 10.3390/jpm10030101

**Published:** 2020-08-25

**Authors:** Laura Le Gall, Ekene Anakor, Owen Connolly, Udaya Geetha Vijayakumar, William J. Duddy, Stephanie Duguez

**Affiliations:** 1 Northern Ireland Center for Stratified/Personalised Medicine, Biomedical Sciences Research Institute, Ulster University, Derry-Londonderry BT47, UK; le_gall-l@ulster.ac.uk (L.L.G.); Anakor-E@ulster.ac.uk (E.A.); Connolly-O4@ulster.ac.uk (O.C.); u.vijayakumar@ulster.ac.uk (U.G.V.); w.duddy@ulster.ac.uk (W.J.D.); 2 NIHR Biomedical Research Centre, University College London, Great Ormond Street Institute of Child Health and Great Ormond Street Hospital NHS Trust, London WC1N 1EH, UK

**Keywords:** oxidative stress, mitochondria dysfunction, axonal transport, autophagy, endocytosis, secretion, excitotoxicity, RNA metabolism, MND

## Abstract

Amyotrophic lateral sclerosis (ALS) is a terminal late-onset condition characterized by the loss of upper and lower motor neurons. Mutations in more than 30 genes are associated to the disease, but these explain only ~20% of cases. The molecular functions of these genes implicate a wide range of cellular processes in ALS pathology, a cohesive understanding of which may provide clues to common molecular mechanisms across both familial (inherited) and sporadic cases and could be key to the development of effective therapeutic approaches. Here, the different pathways that have been investigated in ALS are summarized, discussing in detail: mitochondrial dysfunction, oxidative stress, axonal transport dysregulation, glutamate excitotoxicity, endosomal and vesicular transport impairment, impaired protein homeostasis, and aberrant RNA metabolism. This review considers the mechanistic roles of ALS-associated genes in pathology, viewed through the prism of shared molecular pathways.

## 1. Introduction

Amyotrophic lateral sclerosis (ALS) is the most frequent motor neuron disease (MND), with an estimated ~223,000 patients being affected globally in 2015 [1]. The pathology affects both upper motor neurons (UMN) in the cortex and lower motor neurons (LMN) in the brainstem and spinal cord [2]. Paralysis and death usually occur between three to four years after symptom onset [3], and there are currently no effective treatments to slow disease progression [4]. Approximately 90% of ALS cases are sporadic, while 10% are familial, defined by the occurrence of ALS in more than one family member [5]. Around 30 different genes are linked with ALS [5,6], explaining ~20% of all ALS cases and associated with different molecular functions and disease phenotypes [7], so that the task of understanding the relationships between affected pathways is complex.

To investigate the different molecular pathways affected in ALS, various in vivo models, including drosophila [8,9,10,11], C-elegans [12], zebrafish [13,14,15,16], and rodents [17], as well as in vitro cell models such as patient lymphoblastoid cell lines [18] and hybrid [19,20] or primary murine cell lines, [21] have been developed. Most of these models investigate the pathological effects of mutations to ALS genes, including *Fused in Sarcoma* (*FUS*), *Superoxide dismutase* (*SOD1*), *TAR DNA-binding protein 43* (*TDP-43*), and *Chromosome 9 open reading frame 72* (*C9orf72*) [22,23]. Their study has resulted in numerous cellular and molecular mechanisms being proposed to explain motor neuron death. Mechanisms frequently implicated include: reactive oxygen species (ROS)-associated oxidative stress [24,25,26,27], mitochondrial dysfunction [24], axonal and vesicular trafficking dysregulation [28,29], glutamate-mediated excitotoxicity [30,31,32,33], proteostatic impairments [34,35,36,37,38], and altered RNA metabolism and/or processing [39,40,41,42].

Alteration to one or more of these cellular processes may be present, not only in the motor neurons themselves but, also, in neighboring cell populations, such as glial cells, peripheral inflammatory cells, and muscles, as ALS is increasingly considered a multisystemic disease that culminates in motor neuron death [6,24]. For example, astrocytes and microglia have been implicated in the release of proinflammatory mediators that lead to chronic neuroinflammation and motor neuron toxicity [43]. In addition, the selective overexpression of mutant SOD1 in skeletal muscle was shown to cause mitochondrial abnormalities, induce microglial activation in the central nervous system (CNS), and result in severe muscle atrophy in mice [44].

Consensus is yet to be reached regarding the causal mechanisms involved in the onset and propagation of ALS. The aim of this review is to identify and summarize the different molecular mechanisms implicated in various forms of the disease, including sporadic and familial cases. In doing so, it is hoped that new insights may be gained regarding the role of different pathways across different forms of the disease.

## 2. Oxidative Stress

**Figure 1:**
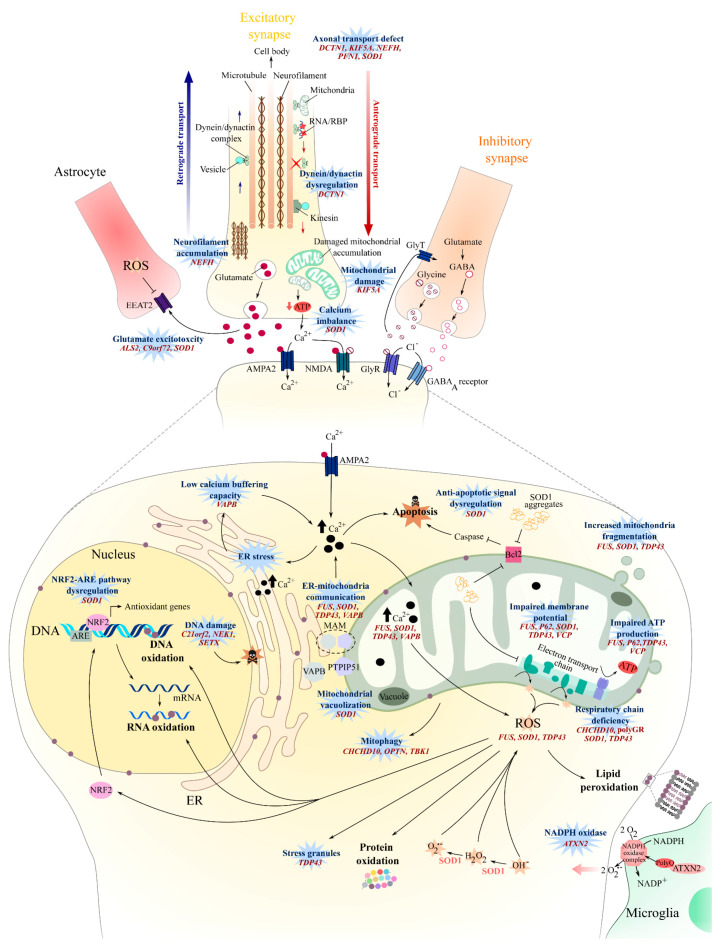
Oxidative stress, mitochondrial dysfunction, axonal transport, and glutamate excitotoxicity in amyotrophic lateral sclerosis (ALS). An increase in oxidative stress can result from defects in detoxifying pathways. Such defects include the loss of SOD1 function, aberrant DNA damage repair machinery, or a decrease in expression of antioxidant genes affecting the NRF2-ARE pathway. Oxidative stress can also be increased by the stimulation of ROS production via increased NADPH oxidase activity or from disrupted mitchondrial respiratory chain activity. Mitochondrial activity can be affected by several ALS mutations, such as those leading to the accumulation of protein aggregates, or to decreased mitochondrial biogenesis and transport, or to increased cytosolic Ca^2+^ (as observed when glutamate receptor activity is stimulated or when the Ca^2+^-buffering capacity is decreased). Consequently a disruption of the mitochondrial respiratory chain will lead to an increase in ROS production and, thus, to an accumulation of oxidized proteins, lipids, DNA, and RNA. Oxidative damage occurring over time may then stimulate apoptotosis and, thus, cell death. Defective axonal transport affects not only the mitochondria but, also, the transport of other proteins and RNA, with consequences on the axon structure and function being accompanied by neurofilament accumulation. Defective glutamate uptake by astrocytes, and/or a defect in glutamate receptor clearance or in AMPA or GABA receptors, can lead to increased Ca^2+^ permeability and can impact the post-synaptic hyperexcitability and mitochondrial function. ARE: antioxidant response element, AMPA2: α-amino-3-hydroxy-5-methyl-4-isoxazolepropionic acid receptor 2, ATXN2: ataxin 2, Bcl2: B-cell lymphoma 2, C9orf72: Chromosome 9 open reading frame 72, C21orf2: Chromosome 21 open reading frame 7, CHCHD10: coiled-coil helix coiled-coil helix domain-containing 10, DCTN1: Dynactin 1, EEAT2: Excitatory amino acid transporter, ER: endoplasmic reticulum, FUS: Fused in Sarcoma, GABA: gamma-Aminobutyric acid, GlyR: glycine receptor, GlyT: glycine transporter, KIF5A: Kinesin heavy-chain isoform 5A, MAM: Mitochondria-associated ER membranes, NEFH: heavy-weighted neurofilaments, NEK1: (NIMA)-related kinase 1, NMDA: N-methyl-D-aspartate receptor, NRF2: Nuclear erythroid 2-Related Factor, PFN1: profilin-I, PTPIP51: Protein tyrosine phosphatase-interacting protein 51, SETX: senataxin, SOD: Superoxide dismutase 1, SPG11: Spatacsin, TDP-43: TAR DNA-binding protein 43, VAPB: vesicle-associated membrane protein-associated protein B, VCP: valosin-containing protein, and ROS: reactive oxygen species.

Oxidative stress results from an imbalance between the production and elimination of reactive oxygen species (ROS) [45], as well as an impaired ability to repair ROS-mediated toxicity [46], and has been of particular interest in ALS pathogenesis ([47] and Figure 1) since the discovery of *SOD1* mutations in familial forms of ALS [48]. Increased levels of oxidized proteins, RNA, DNA, and lipids have been observed in post-mortem tissue from both sporadic and *SOD1* ALS cases [27,49,50], as well as in the cerebrospinal fluid (CSF), serum, and urine of sporadic ALS patients [26].

SOD1 is a major antioxidant enzyme that is ubiquitously expressed and catalyzes radical superoxide anions into molecular oxygen and hydrogen peroxide [51]. Approximately 80 of the 160 *SOD1* mutations reported in ALS are missense mutations that fail to cause a loss of SOD1 activity [52], and many SOD1 mouse models show a progressive, late-onset motor phenotype with concomitant astrogliosis and motor neuron pathology when mutated forms of human SOD1 are overexpressed [17]. Evidence from human samples have shown that there is a 42% reduction in overall SOD1 activity in familial SOD1 patients [53], potentially leading to an imbalance between ROS production and degradation (Figure 1). This imbalance might be exacerbated by the disruption of the NRF2-ARE (Nuclear erythroid 2-Related Factor—antioxidant response element) signaling pathway that is observed in *SOD1* ALS [54], thus affecting the expression of antioxidant proteins [55] (Figure 1). Supporting these hypotheses, oxidative damage such as protein glycoxidation and lipid peroxidation were observed in the motor neurons of the anterior horn from *SOD1* familial ALS (fALS) patients [56] and SOD1^G93A^ mice [57,58].

The generation of ROS could result from the activity of NADPH oxidase in the lipid raft membrane compartment. Interestingly, the *ATXN2* gene encodes the ataxin-2 polyglutamine (PolyQ) protein, and intermediate-length PolyQ expansions (27–33 Qs), which are known to be a significant risk for ALS [59,60,61], can interact with NADPH oxidase and may lead to an increase in ROS production, DNA damage, and mitochondrial distress [62] (Figure 1).

Recurrent oxidative stress and/or mitochondrial dysfunction occurring throughout the life of the cell can lead to DNA damage—damage that can be fixed by activating the DNA damage repair machinery. Several genes known to encode for proteins involved in DNA damage repair [63,64,65] are also associated with ALS: *NEK1* [66], *C21orf2* [67], and *SETX* [68]. These encode for the proteins never in mitosis-A (NIMA)-related kinase 1 (NEK1), cilia and flagella-associated protein 410, and the DNA/RNA helicase senataxin, respectively. Mutations in these genes may therefore increase the susceptibility to ALS as a result of dysregulated DNA damage repair machinery [67,69,70], leading to an impaired ability of motor neurons to cope with oxidative stress, consequently leading to cell death [64,70] (Figure 1). For example, induced pluripotent stem cell (iPSC) motor neurons derived from NEK1^c.2434A>T^-mutated ALS patients exhibit an increased level of DNA damage, as well as a failure to repair DNA double-strand breaks [70]. Primary motor neurons from *SETX*^R2136H^ and *SETX*^L389S^ murine models were unable to cope with induced oxidative stress and showed an increased stress granule formation [71].

Altogether, these studies suggest that oxidative stress might be increased in sporadic and familial ALS patients. Increased oxidative stress may affect mitochondrial function [72], exacerbate endoplasmic reticulum stress [73], and impact protein homeostasis mechanisms [74], ultimately leading to cell damage and neuronal loss.

## 3. Mitochondrial Dysfunction

Mitochondria are key organelles for ATP generation, calcium buffering, and apoptosis regulation [75], and their dysfunction in the dorsal root ganglion cells of sporadic ALS patients has been described previously [76]. Several mechanisms can trigger mitochondrial dysfunction in ALS (Figure 1).

The maintenance of mitochondrial cristae organization is crucial to ensure respiratory chain function [77] and requires cardiolipin, the ATP synthase dimer, and large protein complexes such as the mitochondrial contact site complex (MICOS) and dynamin-like Opa1/Mgm1 [78,79]. The coiled-coil helix coiled-coil helix domain-containing protein 10 (CHCHD10), known to be associated with ALS [23], is suspected to be either part of [80,81] or interact with MICOS [82]. Consequently, mutations in *CHCHD10* result in the loss of mitochondria cristae [80], mitochondria fragmentation [81], and defective mitochondrial repair [80,83] (Figure 1).

Mitochondrial biogenesis can also be directly affected, as observed in *FUS*-mutated conditions [84,85]. While *FUS* encodes for a DNA/RNA-binding protein [86] predominantly localized to the nucleus, mutated forms of FUS can accumulate in the cytosol and possibly become toxic [87,88] and affect the mitochondrial function. For example, the mutated FUS^P525L^ can interact with mitochondrial chaperone proteins and induce mitochondria fragmentation and elevated ROS production [84,85].

Aberrant swollen mitochondria morphology has also been observed in neuronal, non-neuronal cells, and muscle tissue of other fALS cases, such as *SOD1* [24,89] and *C9orf72* [25,89]-mutated ALS patients but, also, in both SOD1^G93A^ and TDP-43^A315T^ murine models [44,90,91]. The aberrant morphology may result from a cascade of events involving the mutated protein aggregates. For example, insoluble mutant SOD1 can aggregate in mitochondria in the spinal cord of SOD1^G93A^ mice [92], causing the formation of vacuoles in the outer- and inter-mitochondrial membrane [93], affecting mitochondrial respiration, energy production, and ultimately, increasing the level of oxidative stress [94] (Figure 1). ALS patients with the SOD1^A4V^ mutation show significant increases in complex I and III activity of mitochondria in the motor cortex [95,96]. The overactivation of complexes I and III increased the production of mitochondrial ROS [97] and may explain the high level of oxidative stress observed in SOD1 mice and patients.

The G4C2 hexanucleotide repeat expansion mutation (HREM) in the *C9orf72* gene explains 40–50% of familial ALS cases and 5–10% of sporadic cases [98,99,100,101]. There are several hypotheses regarding the mechanisms by which this leads to toxicity, and evidence exists for both loss and gain-of-function-mediated toxicity. One hypothesis suggests that the repeat-associated non-AUG (RAN) translation of G4C2 repeats is causal in the expression of toxic dipeptide repeat (DPR) proteins. RAN translation can occur in both sense and antisense reading frames [41], resulting in the production of five different DPRs: glycine-alanine (GA), glycine-arginine (GR), proline-arginine (PR), proline-alanine (PA), and glycine-proline (GP) [38]. Interestingly, the expression of poly-GR results in early abnormalities in the mitochondiral respiratory chain by interacting with ATP5A1, a complex V protein, and leads to its ubiquitination and degradation in *C9orf72* ALS-FTD patients [102]. Mitochondrial dysfunction [103] and an increased oxidative stress [104] are reported in fibroblasts and iPSC-derived astrocytes obtained from *C9orf72* ALS patients (Figure 1).

Nonfunctional and damaged mitochondria can be targeted by NIP3-like protein X (NIX) or PTEN-induced putative kinase protein 1 (PINK1)-E3 ubiquitin ligase parkin, then sequestered into isolation membranes and degraded after fusion with the autophagosome or lysosome [105]. Optineurin (OPTN) and TANK-binding kinase 1 (TBK1) are key actors for mitochondria engulfment [106]. Consequently, ALS mutation in *OPTN* [107] and *TBK1* [23] will affect the mitophagic flux and may lead to an accumulation of nonfunctional mitochondria over time and result in motor neuron death (see [108] for review). Taken together, except for *CHCHD10*, these studies point toward mitochondria dysfunction and damage being a downstream effect of ALS gene mutations that lead to protein aggregations and/or proteostasis dysfunction (see Section 7: Impaired Protein Homeostasis).

In addition, damage to mitochondria and alterations in their functions can disrupt calcium homeostasis, increasing the sensitivity of neurons to glutamate excitotoxicity and the risk of motor neuron damage ([109], Figure 1). Mitochondrial dysfunction can also activate proapoptotic signals [93], such as the caspase-dependent [110] or bcl-2-dependant pathways [93], and might lead to motor neuron degeneration.

## 4. Axonal Transport

Motor neurons have exceptionally long axons, up to 1 m in length, placing extreme demands on cellular physiological functions that rely on the axonal transport of organelles such as mitochondria or of molecules including proteins, lipids, and RNA to and from the synapse [111]. Axonal transport, as well as the conduction of electrical impulses and the maintenance of the axon structure, are heavily regulated processes linked with control of the neurofilament structure [112,113]. In both sporadic ALS ( sALS) and fALS patients, the disorganization of neurofilament networks has been reported [38].

Neurofilaments are neuron-specific intermediate filaments that are stretch-resistant and are major cytoskeleton proteins [114]. They form parallel coiled-coiled heterotetramers composed of light, medium, and heavy-weighted neurofilaments (NF-L, NF-M, and NF-H, respectively) and α-internexin or peripherin [112,114]. Eight heterotetramers form cylindrical structures known as unit-length filaments (ULFs) with the tail domains sticking out [112,114]. A series of ULFs form a filament that matures into neurofilament after a radial compaction of the cylindrical structure [112]. Consequently, variants in the *NEFH* gene affecting the crosslinking properties of the NF-H protein may result in abnormal neurofilament accumulations and in axonal transport defects [115].

Neurofilaments form cross-bridges not only with each other but, also, with actin filaments, actin rings, and microtubules [114], constituing a protein network that might participate to the maintenance of the axon structure [114,116]. Actin polymerization requires the small actin-binding proteins profilin I and II and phosphoinositide islands localized at the membrane [117]. Mutations in the *PFN1* gene encoding profilin-I are associated with ALS [118], and the expression of mutant hPFN1^G118V^ in a murine model resulted in dysregulated actin polymerization [119]. Consequently, the attachement of actin to the microtubules might be affected, probably impacting anterograde and retrograde transport and, thus, leading to an accumulation of fragmented mitochondria and, ultimately, to upper and lower motor neuron death ([119], Section 3 and Figure 1).

Microtubules and motor proteins such as the dynein-dynactin complex [28,120,121] and the kinesins [120,122,123] are involved in the long-distance transport of cellular cargo. Microtubules are composed of dimers of α- and β-tubulin. The alpha tubulin subtype TUBA4A is an ALS-associated protein [124], and ALS-associated mutations of *TUBA4A* lead to microtubule polymerization defects and network destabilization [124].

The dynein-dynactin complex [28,120,121], along with the kinesins [120,122,123], are key drivers of the anterograde and retrograde movements of diverse cargoes along the microtubule cytoskeleton, including organelles, vesicles, neurofilaments, AMPA and GABA receptors, and RNAs. Interestingly, mutations in *dynactin subunit 1 (DCTN1)* affecting the tertiary structure of the dynactin protein and its capacity to bind to microtubules can cause ALS [125]. When the interaction between dynein-dynactin is interrupted by the overexpression of dynamitin, axonal transport is impaired, and mice develop a late-onset motor pathology that recapitulates late-onset progressive ALS [126]. Kinesins form a superfamily of molecular motors that can be divided into three groups [120]. KIF5, a member of kinesin 1-group, is a tetramer with two kinesin heavy chains (KHCs) that contains a motor domain and two kinesin light chains (KLCs) that facilitate connections with cargo. There are three KIF5 isoforms—KIF5A, KIF5B, and KIF5C—all three isoforms being associated with the neuronal function and anterograde transport of proteins and organelles [127]. Mutations in the C-terminal of KIF5A, leading to a loss of function, are associated with ALS [128] and are suspected to disrupt the axonal transport (Figure 1). This hypothesis is supported by the defective axonal transport of mitochondria, the local accumulation of neurofilament, and the reduced axonal growth and survival observed in the primary culture of motor neurons from KIF5A^−/−^ mice [129].

Distal axonal transport is also affected in SOD1^G93A^ mice at an early stage, with an early decrease in kinesin expression in asymptomatic mice, followed by a decrease in dynein expression in older presymptomatic mice [130]. Defective axonal transport may contribute to the accumulation of impaired mitochondria at distal sites ([93], Figure 1), resulting in decreased ATP production and disrupted calcium homeostasisis at the neuromuscular junction, consequently leading to a distal axonopathy in SOD1^G93A^ mice [109,131,132] and *SOD1* patients [24,28]. Kinesin-dynein machineries have been described to be affected in sporadic ALS, where KIF1Bβ and KIF3Aβ, two kinesin-related proteins, were found to be downregulated in motor cortex samples of sporadic patients [133]. However, the expression level of another kinesin-related protein, KIFAP3, is inversely correlated with sporadic ALS patient survival [134].

In conclusion, different mutations associated to ALS can directly alter the architecture and dynamics of the cytoskeleton, affecting the axonal transport machinery. Interestingly, aberrant axonal transport has also been observed in sALS patients and in fALS patients harboring mutations in non-cytoskeletal-related genes. Disrupted transport mechanisms can then affect the mitochondrial metabolism and degeneration (Section 3), as well as protein degradation (Section 7) and RNA transport (Section 8), ultimately leading to motor neuron death.

## 5. Glutamate Excitotoxicity

Glutamate is the most abundant neurotransmitter in the CNS and is released from presynaptic neurons into the synaptic cleft, resulting in the activation of NMDA and AMPA receptors that mediate calcium and sodium influxes in postsynaptic neurons. Excess glutamate may result in the abnormal activation of glutamate receptors, causing an excessive influx of Ca^2+^ in the postsynaptic neuron (Figure 1), which leads to extreme neuronal firing [135], resulting in excitotoxicity, which is potentially implicated in a number of pathological conditions, including multiple sclerosis [136], Parkinson’s diasease [137], and ALS [138,139]. Glutamate excitotoxicity is thought to occur as a result of defective glutamate uptake and transport mechanisms that lead to excessive neuronal Ca^2+^ intake, aberrant Ca^2+^ homeostasis, downstream mitochondrial dysfunction, and increased ROS production [140,141] (Figure 1). Glutamate-gated AMPA receptors are abundant in human and animal motor neurons [142,143] and are made up of four subunits, GluA1 to GluA4 (also GluR1–4) [144]. The overactivation of AMPA receptors has been shown to result in hindlimb paralysis and motor neuron degeneration in wild-type rats, highlighting the susceptibility of motor neurons to Ca^2+^ dysregulation [145]. The Ca^2+^ permeability of AMPA receptors is mediated by the presence of the GluA2 subunit, the absence of which, in addition to impaired transcriptional editing at the Q/R site, confers increased AMPA Ca^2+^ permeability [146]. Interestingly, spinal motor neurons have been reported to display a reduced expression of GluA2 relative to dorsal horn neurons from the same region, providing some explanation for the selective susceptibiltiy of motor neurons in ALS [147], and GluA2 transcriptional editing has been found to be impaired in motor neurons of sporadic ALS patients relative to controls [148]. Furthermore, evidence suggests that GluA2 editing is also impaired in ALS oculomotor neurons, despite their spared function in disease. However, spared functionality has been hypothesized to be the result of increased Ca^2+^-binding proteins and, in particular, parvalbumin, which is highly abundant in oculomotor neurons and present at low levels in spinal motor neurons [149]. GluA2 transcriptional editing into the Ca^2+^ impermeable subunit is mediated by adenosine deaminase acting on RNA 2 (ADAR2) activity [150,151]. A reduced ADAR2 expression has been reported in sporadic ALS patients and has been shown to result in an increased aggregation of TDP-43 in spinal motor neurons [152]. Taken together, the evidence suggests that a decreased GluA2 expression and impaired transcriptional editing in spinal motor neuron AMPA receptors is a contributing factor to the increased uptake of Ca^2+^ and the downstream susceptibility to excitotoxicity in ALS (Figure 1). Moreover, the finding that AMPA receptor dysfunction can result in the aggregation of misfolded TDP-43 is an important finding for linking ALS pathology with the glutamate excitotoxicity hypothesis.

In addition to dysregulated GluA2 subunit function, research has reported dysfunctional glutamate transport mechanisms in ALS. Under normal physiological conditions, glutamate at the synaptic cleft is cleared by the excitatory amino acid transporter (EAAT2), which functions to maintain low levels of extracellular glutamate and, thus, prevent excessive increases in intracellular Na^+^ and Ca^2+^ levels [89,153]. EAAT2 is found primarely on the synaptic processes of astrocytes, and the loss of EAAT2 has been reported to induce increased extracellular levels of glutamate and cause motor neuron toxicity and muscle paralysis in animal models [154], whilst the pharmalogical stimulation of EAAT2 was found to rescue motor neuron degeneration and delay paralysis in SOD1^G93A^ mice [155,156]. Abnormalaties in EAAT2 have been suggested to occur post-translationally after a post-mortem research highlighted no differences in EAAT2 mRNA expressions between sporadic ALS patients and controls, despite a 95% decrease in protein levels in sALS subjects [157]. Further support of EAAT2 loss and its implications in ALS were reported by a separate group who demonstrated a reduced EAAT2 immunoreactivity in anterior horn cells of sporadic ALS and lower motor neuron disease patients relative to healthy controls [158]. Together, these studies highlight the role of dysfunctional glutamate uptake and transport mechanisms in sporadic cases of ALS (Figure 1).

Excitotoxicity has also been associated with genetic forms of the disease. *SOD1* mutations have been implicated in the glutamate excitotoxicity hypothesis, and research has demonstrated an increased glutamate release [159], as well as motor neuron and inter-neuron hyperexcitability two to three months prior to motor neuron degeneration and phenotype onset in SOD1^G93A^ mice [160]. *SOD1* mutations have also been shown to reduce the expression of astrocytic GluA2 in vitro and in vivo, thereby diminishing their ability to protect against motor neuron excitotoxicity [21]. In patients, the deterioration of neuronal dendrites was observed in sporadic and familial ALS cases, but not in healthy or Alzheimer’s disease controls, leading to the suggestion that ALS is a synaptopathy [161], which is perhaps attributable to the excessive levels of glutamate observed in the CSF of patients [138,139]. Indeed, metabolomic analyses suggest that ALS patients show elevated serum levels of glutamate [32], and there is evidence that sporadic and familial ALS cases show heightened levels of cortical excitablity, which can be detected even in the presymptomatic stages in familial *SOD1* mutation carriers [162]. However, other studies have failed to find evidence for elevated glutamate levels in ALS patients [163,164,165]. *C9orf72* has also been implicated in the glutamate excitotoxicity hypothesis after iPSC motorneurons from ALS patients were found to have impaired autophagosome formation and aberrant accumulations of glutamate receptors [166,167,168]. This has been supported in vivo with *C9orf72* knockout mice showing GluR1 upregulation in the hippocampus and a greater susceptibility to excitotoxicity compared to controls [166]. In addition, *C9orf72* knockout mice demonstrated a complete loss of SMCR8 [169], a protein that functions in a complex with C9orf72 and WD40 repeat domain 41 (WDR41) to regulate membrane trafficking and autophagy [170]. The concomitant abnormalities in autophagy and aberrant accumulations of GluR1 has led to the hypothesis that *C9orf72* loss-of-function leads to an impaired clearance of excess glutamate receptors, which, in turn, results in a greater glutamate uptake and increased susceptibility to excitotoxicity (Figure 1). *C9orf72* patients have also been reported to demonstrate elevated glutamate levels in their cerebropsinal fluid (CSF), which has been hypothesized to occur as a result of DPR-mediated splicing defects to EAAT2 and subsequent impairments in glutamate clearance [168]. Research has also implicated ALS2 in the glutamate exicitotoxicity hypothesis by virtue of its interaction with Rab5 and the endosomal pathway. ALS2^-/-^ knockout mice have been reported to show significant increases in glutamate receptor degradation, including GluA2 [171], the loss of which is believed to contribute to excitotoxicity and motor neuron degeneration. Similarly, *ALS2*^−/−^ spinal motor neurons were found to be more susceptible to glutamate excitotoxicity as a result of reduced GluA2 at the synapses of neurons, which was attributed to an altered glutamate receptor interacting protein 1 (GRIP1) function, caused by the genetic loss of *ALS2* [171].

Although there is evidence to suggest the presence of glutamate transport and uptake defects in both sporadic and familial cases of ALS, it is unclear how these defects lead to the specific deterioration of motor neurons in disease. Furthermore, it has been 25 years since the approval of the antiglutamatergic drug riluzole for the treatment of ALS, yet there is no understanding as to why it, as well as other antiglutamatergics, including gabapentin, memantine, and ceftriaxone, fail to delay symptom progressions in ALS by more than ~three months [172,173].

## 6. Endosomal Pathway and Vesicle Secretion

Extracellular vesicles encompass different types such as apoptotic vesicles, microvesicles, and exosomes, all of which can affect the functionality of the recipient cells [174,175,176,177]. The last decade has seen several investigations into the relevance of exosomes to ALS, either in propagating the disease or as biomarkers [6,178,179,180,181]. In the ALS context, exosomes secreted by astrocytes, neurons, or microglia are suspected to carry neurotoxic elements such as mutated SOD1 or C9orf72-derived DPR and to be responsible for motor neuron death [178,179,182]. Interestingly, sporadic muscle cells present an accumulation of vacuole and multivesicular bodies in their cytosol, suggesting that vesicle trafficking is disrupted in these cells ([183] and personal data) and that extracellular vesicle secretion might have an important role in ALS.

**Figure 2:**
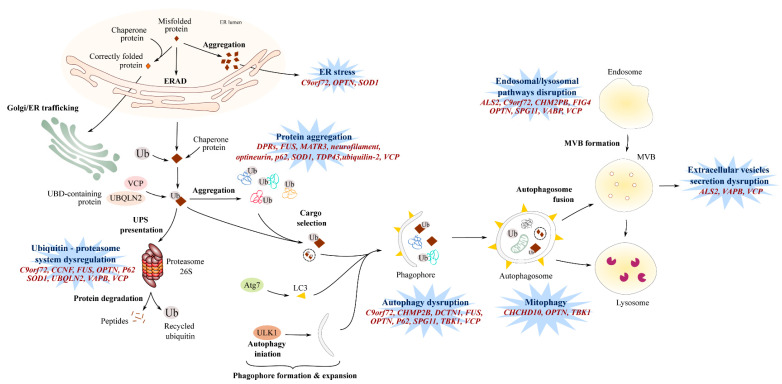
Protein homeostasis dysregulation. Dysregulated protein homeostasis is mediated by multiple pathways encompassing defects in autophagy, the dysregulated ubiquitin-proteasome system (UPS), endo-lysosomal pathway disruptions, or endoplasmic reticulum (ER) stress. The presence of misfolded proteins activates endoplasmic reticulum-associated protein degradation (ERAD), leading to proteasome-mediated degradation to avoid misfolded protein accumulations in the ER lumen and subsequent ER stress. Several ALS-associated gene mutations induce proteasome-mediated toxicity via the sequestration of ubiquilin and chaperone proteins involved in the UPS pathway. The proteolytic activity of the proteasome has also been demonstrated to be targeted by gene mutations in ALS. The autophagic pathway involves the formation and maturation of phagophores that engulf selected transported-cargo and form autophagosomes. Fusion with the lysosome enables the degradation of autophagosome contents. Defects in autophagy initiation and expansion, dysregulated phagophore formation, and/or impaired cargo transport are observed in ALS patients. Mutations in ALS-associated genes also cause defects in mitophagy, a specific form of autophagy. Defects in the endolysosomal have been associated with ALS gene mutations, including defective endolysosomal trafficking and altered lysosomal hoemostasis and degradation. Defects in the autophagy/lysosomal pathway may affect vesicle secretion. Genes implicated in dysregulated protein homeostasis are indicated in red. ER: endoplasmic reticulum, ERAD: endoplasmic reticulum-associated protein degradation, ALS2: Alsin, C9orf72: Chromosome 9 open reading frame 72, CCNF: Cyclin F, CHCHD10: coiled-coil helix coiled-coil helix domain-containing 10, CHMP2B: chromatin-modifying protein 2B, DCTN1: Dynactin 1, FIG4: Phosphoinositide 5-phosphatase, FUS: Fused in Sarcoma, MATR3: matrin 3, MVB: Multivesicular bodies, OPTN: Optineurin, SOD1: Superoxide dismutase 1, SPG11: Spatacsin, TDP-43: TAR DNA-binding protein 43, TBK1: TANK-binding kinase-1, UBQLN2: Ubiquilin-2, VAPB: vesicle-associated membrane protein-associated protein B, and VCP: valosin-containing protein.

Exosome biogenesis requires the formation of inward buds in the multivesicular body (MVB), followed by their fission and release as vesicles into the MVB lumen. The generation of intraluminal vesicles can be either Endosomal Sorting Complex Required for Transport (ESCRT)-dependent or ESCRT-independent. The ESCRT is composed of four complexes—ESCRT-0, ESCRT-I, ESCRT-II, and ESCRT-III—each complex acting one after the other to form intraluminal vesicles. Interestingly, charged multivesicular protein 2B (CHMP2B) is a component of ESCRT-III involved in the processing of cargo into intraluminal vesicles and is associated with ALS [184]. The dysfunction of ESCRT-III may lead to abnormal and dysmorphic endosomes [185] (Figure 2).

Endocytosis and vesicle trafficking from one cellular compartment to another are regulated by small Rab GTPases [186]. For example, Rab5 is associated with the formation of early and late endosomes, while Rabs 11, 35, and 27 have direct roles in exosome biogenesis and secretion. Alsin is an ALS-associated protein [187] and is a guanine nucleotide-exchange factor involved in endosome motility and fusion with the lysosome [171]. Consequently, an absence of alsin expression in hippocampal neurons leads to an accumulation of Rab5-positive vesicles and an enhanced lysosome-mediated degradation, suggesting an enhanced degradation of endosomal vesicles [171], thus probably affecting the production and secretion of exosomal vesicles.

The C9orf72 protein structure presents some similarities with the Differentially Expressed Normal versus Neoplastic (DENN) guanine nucleotide exchange factor and, thus, may activate Rab proteins [101] such as RAB8A and RAB39B [188]; Rab1a [34]; or Rabs 1, 7, and 11 [189], which are associated with autophagy and vesicle-trafficking processes [190,191,192], as well as exosome biogenesis and secretion [186]. In *C9orf72* knockdown cell lines [189], a *C9orf72* knockout murine model [193], and in ALS patient fibroblasts and iPSC-derived motor neurons [194], transgolgi and endosomal trafficking were reduced, a defective autophagy pathway was observed [34,194], and exosomal secretion was affected [194].

Vesicle-associated membrane protein-associated protein B (VAPB) is involved in vesicle trafficking between the endoplasmic reticulum and the golgi apparatus [195,196]. Interestingly, VAPB has been described to interact with RAB7 and colocalize with CD63 [197], two proteins involved in late-endosome formation and exosome biogenesis [186]. However, the impact of ALS-associated VAPB mutation in exosome biogenesis and secretion still needs to be investigated.

Multivesicular body formation is at a crossroad between the autophagy (Figure 2) and secretion pathways, and an autophagic failure may lead to cell secretion [198]. The *VCP* gene is associated with ALS [199] and encodes for a valosin-containing protein, an ubiquitous AAA+ ATPase that interacts with clathrin to form early endosomes but, also, with the autophagy pathways [200]. In this context, a mutation in a valosin-containing protein (VCP) may affect the endosomal pathway, and one can hypothesize that it has an impact on the formation and secretion of exosomes. Other gene mutations associated to ALS, such as protein polyphosphoinositide 5-phosphatase (FIG4) [201,202] or spastacsin (Spg11) [203,204], are associated with the blockade of lysosomal clearance (see Section 7)—the blockade of which could potentially lead to vesicle secretion [198]. However, futher investigations related to exosome pathways in ALS in vivo are needed.

## 7. Impaired Protein Homeostasis

Protein aggregates positive for TDP-43 [36,205], neurofilament [41], FUS [87], or SOD1 [206] are observed in the vast majority of ALS patients, with TDP-43 being present in as many as 98% of sporadic and familial cases [207], meaning that the presence of such aggregates is widely regarded as a hallmark feature of ALS pathology. These deposits can occur in the cytoplasm of neurons [208] and skeletal muscle [99,209], and their presence is highly suggestive of an imbalance between protein synthesis and degradation pathways (Figure 2).

### 7.1. Proteasome and Autophagic Degradation Pathways

In the late 1960s and early 1970s, the presence of protein inclusions in the anterior horn cells of sporadic and familial ALS patients was described [210,211,212]. Later, these inclusions were found to be ubiquitin positive [35], and SOD1 was the first ALS-associated protein found to be immunoreactive within the inclusions of familial patients [213]. Subsequently, ubiquinated inclusions have often been found to be immunoreactive for the ubiquitin-binding protein p62 [214], and up to 98% of sALS and fALS cases show inclusions that are TDP-43-positive [215], with the exception being *SOD1* [216] and *FUS* [216] patients who do not demonstrate TDP-43 inclusions but do demonstrate SOD1 and FUS immunoreactive inclusions. Other ALS proteins that have been implicated in the formation of cytoplasmic inclusions include optineurin (OPTN) [107], ubiquilin 2 (UBQLN2) [217], dynactin 1 (DNCT1) [218], valosin-containing protein (VCP) [219], and matrin 3 (MATR3) [220]. Studying the structure of the main proteins SOD1, FUS, and TDP-43 helped to unravel potential mechanisms involved in protein misfolding and self-propagation within the cells and in surrounding cells. SOD1 is a stable homodimer, thanks to the intrasubunit disulfide bond and its ability to bind zinc and copper. However, a reducing and metal-poor intracellular environment or mutations [221,222,223,224,225,226,227] can abolish these features and destabilize SOD1, leading to the formation of aggregates and amyloid fibril structures [228,229,230,231] that can self-propagate in vitro [229,230]. FUS and TDP-43 proteins possess a low complexity domain that presents similarities with yeast prions [209] and can form large aggregates and amyloid fibril structures [209,229,232,233]. Interestingly, mutated forms of FUS and TDP-43 can induce the misfolding of wild type forms of FUS and TDP-43, respectively [229], and have also been shown to induce the misfolding of wild type forms of SOD1 in vitro [234]. Altogether, these studies suggest a potential mechanism for the self-propagation of misfolded proteins in vitro—misfolded proteins that can potentially be transferred from cell to cell via secreted vesicles, thus propagating the misfolding mechanism to neighboring cells (see Section 6, [178,229,235]). The presence of these protein aggregates has been suggested to impair the proteasome and autophagic degradation pathways and could be key mediators in ALS pathogenesis [38,236,237] (Figure 2).

Dysregulation of the Ubiquitin–Proteasome System (UPS) in ALS patients was first suspected following the identification of mutations in genes encoding ubiquilin 2 [238] and VCP [199], two proteins involved in protein clearance via the ubiquitin-proteasome pathway [239]. Mutations in *OPTN* [240] and *SQSTM1/P62* [241] were then identified, and following this, *SOD1* [236], *VABP* [242], *C9orf72* [25,208], and *CCNF* (cyclin F) [243] mutations were all reported to reduce UPS activation. Ubiquitin-positive inclusions were observed in post-mortem neuronal and muscular tissues of fALS and sALS patients [35] and, more specifically, in *C9orf72* patients [244]. Similarly, SOD1 [245], FUS [87], ubiquilin 2 [246], and C9orf72-derived DPR proteins [247] can generate toxic aggregates positive for some proteasome components [248]. These ubiquitin-positive inclusions can also contain and ”trap” nonmutated forms of SOD1 [48], TDP-43 [205], optineurin [107], and ubiquilin 2 [249], thus exacerbating the already disrupted cellular homeostasis in ALS.

The degradation of ubiquitinated proteins through the autophagy/lysosomal pathway occurs in four steps: (1) the initiation and extension of the bilayer vacuole into phagophores; (2) the transport of selective cargoes (including ubiquitinated proteins, dysfunctional mitochondria, and protein aggregates); (3) the maturation into autophagosomes; and (4) fusion with low pH lysosomes to form autolysosomes where degradation of the cargoes can proceed [250].

The fusion of the endosome with the lysosome for degradation is an tightly regulated event [251] involving the protein polyphosphoinositide 5-phosphatase, FIG4 [252]. Deleterious mutations in *FIG4* in ALS leads to abnormal lysosomal storage [201,202]. Spastacsin is also involved in lysosomal clearance, and the absence of *Spg11* expression impaired the lysosomal-autophagy pathway and is accompanied by an accumulation of lipid within the lysosomes [203,204]. Nonfunctional TBK1 [253] and p62 [254] inhibit the transport of targeted cargoes toward the autophagosome. Interestingly, impaired autophagosome maturation was also observed in ALS cells mutated for *FUS* [255], *VCP* [89], *CHMP2B* [184], and *OPTN* [256]. The importance of autophagy can be observed in studies that stimulate autophagic activation in the presence of ALS mutations. For instance, the stimulation of autophagy in murine and human iPSC-derived neurons expressing *TARDBP* mutations demonstrated a greater clearance of TDP-43 aggregates relative to nonstimulated cells and resulted in improved motor neuron survival [257].

*C9orf72* mutations can interfere with the autophagy pathway at several levels. When *C9orf72* expression is abolished or decreased as suggested by the haploinsufficiency hypothesis [258,259], autophagy is inhibited [34,189,193,194], leading to simultaneous increases in the number of cytoplasmic inclusions immunoreactive for ubiquitin, p62, and TDP-43 [34,101,260]. The impairment of autophagy in *C9orf72* cells may also result in the accumulation of cytotoxic DPR proteins encoded by the G4C2 HREM and, ultimately, lead to neuronal loss [261]. Similarly to cells expressing *TARDBP* mutations [257], the stimulation of autophagy abolished the accumulation of poly-DPR proteins and neuronal toxicity [261].

Altogether, these studies illustrate the importance of autophagy for the efficient clearance of misfolded and aggregated proteins and is indicative of the underlying impairments in proteostatic mechanisms that mediate ALS physiopathology (Figure 2).

### 7.2. Endoplasmic Reticulum Stress

During the formation of misfolded proteins, the unfolded-protein response (UPR) may be initiated to transport defective proteins to the endoplasmic reticulum (ER), where ER-resident chaperones will properly fold the protein [262]. The accumulation of misfolded proteins in the ER activates the ER stress response pathway, also known as the endoplasmic reticulum-associated protein degradation (ERAD) pathway. The ERAD pathway involves the translocation of misfolded proteins from the ER lumen to the cytosol, where they undergo ubiquitination and degradation through the ubiquitin-proteasome pathway [262]. In ALS patient cells, mutated SOD1 agregates were observed in ER and colocalized with UPR markers, leading to an increase in ER stress [263] by interacting with ER stress response proteins and inhibiting their function in the ERAD response [236]. The presence of poly (GA) aggregates, observed in neuronal post-mortem *C9orf72* ALS patients, can inhibit proteasome activity and induce ER stress, which can be abolished when using ER stress inhibitors such as salubrinal and tauroursodeoxycholic acid (TUDCA) [208]. Concordantly, the cerebropsinal fluid (CSF) of sporadic ALS patients displays an accumulation of ER stress markers [263], and when healthy neurons were exposed to patient CSF, the ER became fragmentated and caspase-dependent apoptosis was activated, suggesting an increase in ER stress [264].

Vesicle-associated membrane protein-associated protein B (VAPB) is localized in the endoplasmic reticulum membrane and has a key role in vesicle trafficking between the endoplasmic reticulum, golgi apparatus, and the nuclear envelope [195,196,197]. The VAPB^P56S^ mutation associated with ALS leads to a misfolded protein that accumulates in the ER [265] and can cause a defect in nuclear envelope protein transport, leading to an aberrant nuclear envelope structure [266]. Interestingly, the accumulation of VAPB has also been observed in the endoplasmic reticulum of peripheral blood mononuclear cells of sporadic ALS [267].

Optineurin is a TBK1 partner and is involved in mitophagy (Section 3). When the association of optineurin with myosin VI is disrupted, as osberved in fALS cases associated with *OPTN* mutations, optineurin is diffused in the cytosol of neuronal cells and results in ER stress and Golgi apparatus fragmentation, as well as an inhibition of the autophagy pathway ([256], Figure 1 and Figure 2).

Altogether, these studies suggest that protein degradation could be directly and indirectly affected in ALS, causing protein aggregation that leads, in turn, to the disruption of the function of organelles such as nuclei (Section 7.2) and mitochondria (Section 3) or to the blockage of lysosomal activity that can potentially affect cell-cell communication (Section 6).

## 8. Aberrant RNA Metabolism

**Figure 3:**
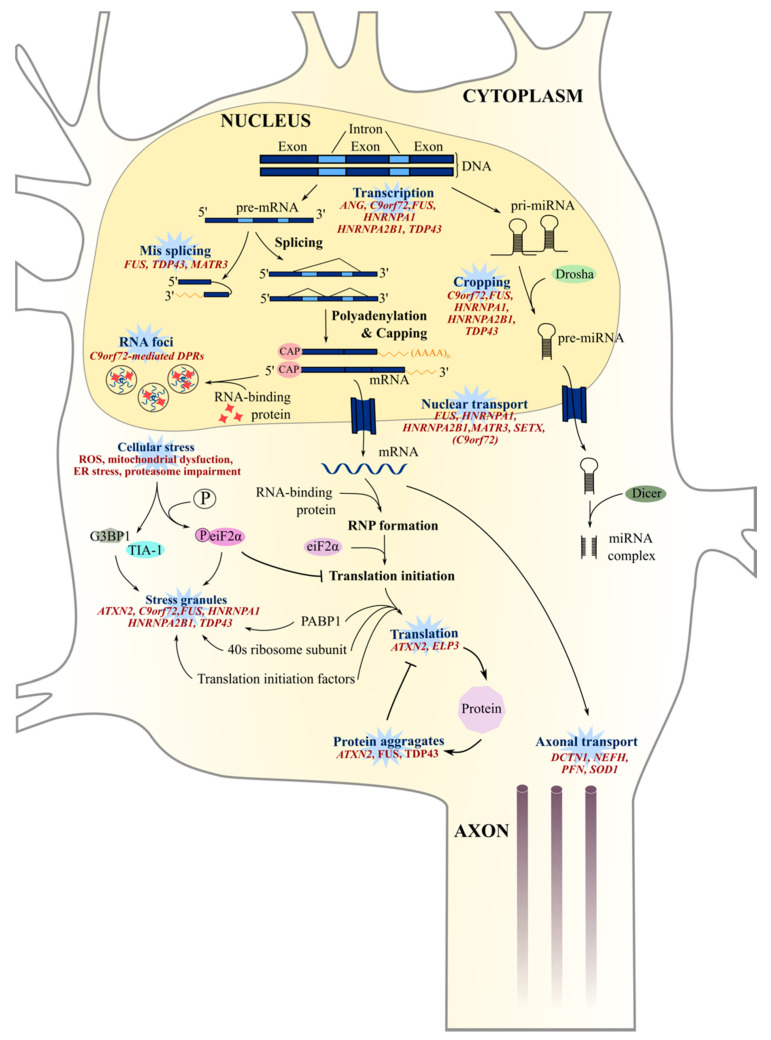
RNA and miRNA biogenesis defects in ALS. Many processes in RNA and miRNA pathways are disrupted in ALS patients, including transcription defects, alternate splicing events, miRNA biogenesis, and nucleus-cytosol transport impairment. RNA metabolism defects are particularly relevant in ALS pathogenesis, since TDP-43 and FUS are both well-known ALS-associated genes involved in RNA processing. Both FUS and TDP-43-mutated proteins mislocalize to the cytoplasm of ALS motor neurons, leading to a probable loss and/or toxic gain-of-function of these proteins. ANG: Angiogenin, ATXN2: Ataxin-2, C9orf72: Chromosome 9 open reading frame 72, DCTN1: Dynactin 1, eIF2α: Eukaryotic translation initiation factor 2A, ELP3: Elongator protein 3, FUS: Fused in Sarcoma, G3BP1: Ras GTPase-activating protein-binding protein 1, hNRNPA1: Heterogeneous nuclear ribonucleoprotein A1, hnRNPA2B1: Heterogeneous nuclear ribonucleoprotein A2B1, MATR3: matrin 3, NEFH: Neurofilament heavy subunit, PABP1: Polyadenylate-binding protein 1, PFN1: Profilin, SETX: Senataxin, SOD1: Superoxide dismutase 1, TDP-43: TAR DNA-binding protein 43, and TIA-1: TIA1 Cytotoxic Granule-Associated RNA-Binding Protein.

FUS [86,268] and TDP-43 [269] are RNA-binding proteins involved in multiples steps of RNA metabolism. In ALS patients, mutations in both genes give rise to the translation of proteins frequently mislocalized to the cytoplasm [87,88,270] and, subsequently, result in downstream complications that affect RNA-processing mechanisms. Dysregulated RNA metabolism is another key feature of ALS pathogenesis and includes transcription defects, alternate splicing changes, miRNA biogenesis, stress granule formation, and RNA nucleocytoplasmic transport (Figure 3).

### 8.1. RNA Splicing and Translation

Given the large number of possible protein-protein interactions between *FUS* or *TDP-43* and their partners, it is easy to expect alterations to important RNA-processing mechanisms in ALS patients [89]. *FUS* and *TDP-43* also regulate the expression of multiple proteins involved in neuronal physiology, including components of the synaptic plasticity pathways [39,271,272] and dendritic branching processes [272,273,274]. In addition, the HREM in *C9orf72* generates repeat RNA and RNA foci, which repress the gene expression of RNA metabolism regulators (such as *hnRNPA3*) [275] or sequester TDP-43 [275,276] and FUS [275] proteins and, thus, indirectly inhibits the transcription of RNA metabolism-associated genes. Similarly to *C9orf72*-mediated RNA-processing defects, *FUS* mutations have been associated with major transcriptional defects [268].

Ataxin-2 is a polyglutamine (polyQ) protein that is involved in mRNA translation, and it interacts with RNA-binding proteins such as TDP-43 and FUS [277]. In ALS spinal cords, ataxin 2 exhibited significant cytoplasmic accumulation and enhanced the toxicity of TDP-43 in Drosophila via RNA binding [59].

*TDP-43*, *FUS*, *hnRNPA1*, *hnRNPA2B1*, and *MATR3* are associated with ALS and involved in pre-mRNA processing [220,278,279,280,281]. Consequently, *TDP-43* knockdown in murine tissues results in the alternate splicing dysregulation of numerous mRNA transcripts [42,282], and the loss-of-function of *FUS* also induces many splicing defects [39], suggesting important alternate splicing events in ALS patients (Figure 3). These downstream complications are not surprising given the ability of the protein FUS to sequester numerous components of the splicing process, such as key splicing factors [283] U1 snRNP and U11/U12 snRNPs [274,284], which are involved in minor intronic splicing. Alternative splicing changes have been identified in neuronal genes involved in cytoskeleton organization, axonal growth, and guidance in *FUS*-mutated ALS patients [285,286], and, interestingly, axonopathy and axon retraction occur in the early stages of ALS [287].

ALS-linked MATR3^S85C^ and MATR3^P154S^ mutations were observed to affect Matrin 3 interactions with the TRanscription and EXport (TREX) protein complex, altering the global nuclear export of mRNA [288]. As a result, mRNA is sequestered within the nucleus, causing export defects of TDP-43 and FUS mRNA [288], which may affect mRNA splicing directly [289] and indirectly [278] (Figure 3). Consequently, as observed in the *MATR3^S85C^* murine model, dysfunctional MATR3 may lead to astrocyte and microglia activation and result in spinal motor neuron degeneration [290].

The *ELP3* gene encodes for elongator protein 3, a histone acetyltransferase subunit of the RNA polymerase II elongator complex responsible for RNA translation (Figure 3). Mutations in the *ELP3* gene are associated with ALS [291,292] and result in the shortening and abnormal branching of motor neurons, as observed in *ELP3* knockdown in zebrafish embryos [291], and altered tRNA modification, triggering proteome impairment and the subsequent aggregation of susceptible proteins [292].

Angiogenin, encoded by the hypoxia-inducible gene *ANG*, is a member of the pancreatic ribonuclease superfamily [293] and, as well as angiogenesis, is also involved in ribosomal biogenesis [294,295]. Defects in this protein are associated with the impairment of its nuclear localization and diminished ribonucleolytic activity [295] (Figure 3), both of which are essential for normal ANG functioning and motor neuron viability.

Together, these findings suggest that RNA processing is a key pathway affected in ALS, either due to mutations directly affecting proteins involved in RNA processing or as a consequence of protein aggregations.

### 8.2. RNA Foci

Sense and antisense RNA generated from the bidirectional transcription of G4C2 repeats have been proposed to induce a toxic gain-of-function in ALS *C9orf72* patient cells by forming RNA foci that may sequester RNA-binding proteins, thus disrupting RNA metabolism and processing in cells [101] (Figure 3) widely throughout the central nervous system [296,297]. Both sense and antisense RNA foci are frequently observed in nucleoli, with antisense RNA foci being denser [296]. In addition, the antisense RNA foci correlate with TDP-43 aggregation in the cytosol of *C9orf72* motor neurons [296,297]. An in situ hybridization of post-mortem *C9orf72* ALS tissue revealed that 78.7% of the neurons and 24.9% of the glial cells in the motor brain and spinal cord regions were positive for antisense RNA foci [297]. Interestingly, extra-motor brain regions also show a high percentage of cells positive for antisense RNA foci, with 89.4% of neurons and 46.1% of glia being positive [297].

### 8.3. Epigenetic Modulation

Epigenetic mechanisms such as microRNA regulation maintain cell type and tissue identity and may be involved in the onset and progression of neurodegenerative diseases, including ALS. The decreased expression of miRNAs, including miRNAs let-7e, miR-148b-5p, miR-577, miR-133b, and miR-140-3p, were observed in post-mortem spinal cords of sporadic ALS patients [298], suggestive of impairment in the genes and pathways associated with miRNA biogenesis, neuroinflammation, and apoptosis.

Interestingly, the class II ribonuclease, Drosha, interacts with TDP-43, FUS, and *C9orf72*-mediated DPRs [299,300,301], while the Dicer enzyme interacts with TDP-43 protein [301] and FUS can interact with pri-miRNA [302] (Figure 3). Consequently, mutated *TDP-43* may impair the post-transcriptional regulation of miRNAs and lead to an altered expression of miR-132-3p and miR-132-5p (involved in the regulation of neuronal outgrowth [301]), miR-143-3p and miR-143-5p (involved in myoblast cell differentiation [303]), miR-558-3p (involved in neurofilament stability [304]), and miR-574-3p (associated with stroke [305]) [301]. Similarly, the downregulation of FUS in a neuroblastoma cell line had a considerable impact on the biogenesis of miRNAs, with an altered expression of miR-9, miR-125b, and miR-132 implicated in neuronal differentiation, activity, and function [302], while mutated *FUS* affected the expression levels of miR125 and miR192 [302], which are involved in early neural conversion [306] or senescence [307].

Altogether, these findings are consistent with defective miRNA processing in ALS patients, which may affect downstream pathways with an impact on motor neuron survival.

### 8.4. Stress Granules and Nucleocytoplasmic Transport

In response to stressful conditions, RNA granules, also known as stress granules, are generated and can recruit FUS and TDP-43 [41,308]. Mutations in *FUS* and *TDP-43* can increase the persistence of stress granules in the cytoplasm, resulting in a possible toxic gain-of-function [237] by inhibiting mRNA translation and, thus, contributing to the progression of ALS pathology (Figure 3). The heterogeneous nuclear ribonucleoprotein particle proteins hnRNPA1 and hnRNPA2B1 are RNA-binding proteins and binding partners of TDP-43 and are involved in RNA processing, including miRNA maturation, the nucleocytoplasmic transport of mRNA, and RNA metabolism [300,309]. Mutations in the prion-like domains of hnRNPA2/B1 and hnRNPA1 increase fibril formation and aggregation potential, as well their hyperassembly into stress granules [310,311]. Stress granules are then targeted to the lysosome by the autophagic machinery involving VCP. Indeed, the pharmacological inhibition or RNAi knockdown of VCP is accompanied by reduced stress granule clearance, while Hela cells expressing VCP^A232E^ and VCP^R155H^ mutations showed a constitutive appearance and accumulation of stress granules containing TDP-43 [312]. Concordantly, the ALS-VCP mutation is accompanied by an increase in stress granules [313].

In *C9orf72* patients, stress granules are also involved in the sequestration of proteins required for effective nucleoplasmic transport, such as RAN GAP [40], or importing and exporting proteins [40,41]. The impairment of the nucleoplasmic transport of molecules in *C9orf72* ALS cells is controverisal. Indeed, while some studies observed that newly formed DPRs such as poly-PR can bind to nuclear pore transporters, thereby impairing the subsequent translocation of molecules [314], other studies did not observe any disruption in the nucleocytoplasmic transport with poly-GR or poly-PR [314]. However, with the expression of poly-GA, defects were observed both in import and in export in a SH-SY5Y cell line and in iPSC-derived motor neurons, respectively [101].

## 9. Concluding Remarks

**Figure 4:**
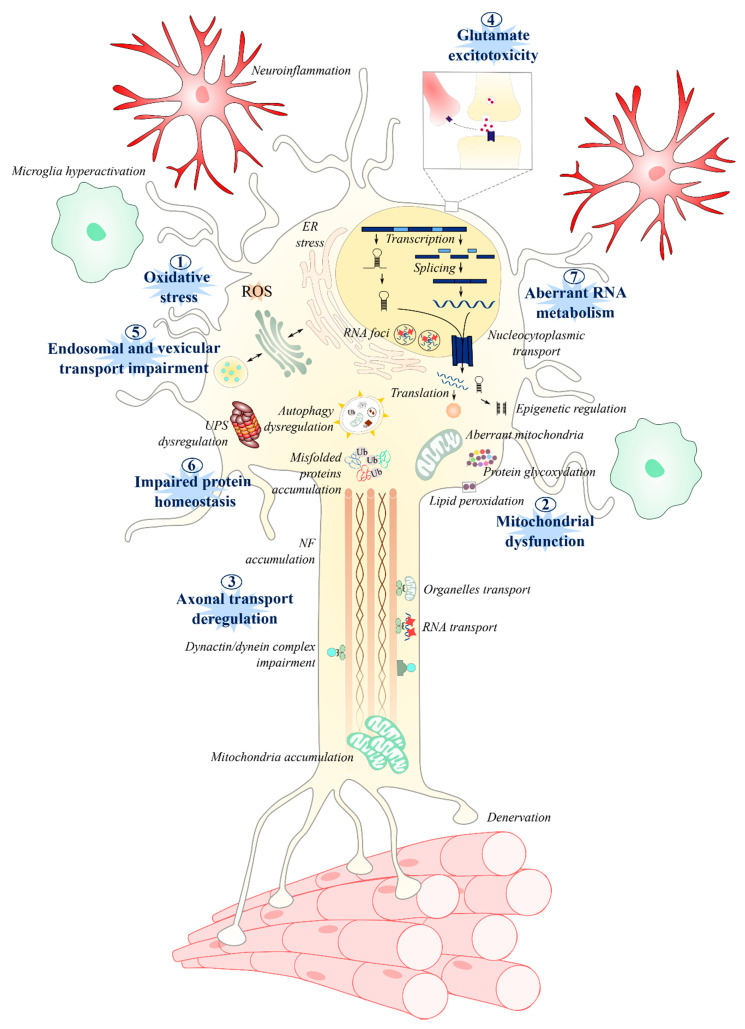
Summary of the different molecular and cellular mechanisms involved in ALS pathogenesis. Among the most studied and well-established pathways are: oxidative stress, mitochondrial dysfunction, axonal transport, glutamate excitotoxicity, endosomal and vesicle secretions, protein homeostasis, and RNA metabolism. One pathway may lead to another, exacerbating the disruption of cellular homeostasis. The disruption of these pathways can lead to microglia activation, neuroinflamation, astrocytosis, and, ultimately, to motor neuron death and muscle denervation.

Over 150 years have passed since ALS was first reported by Charcot, and still, the etiology of the disease remains elusive. Although research is progressing and genetic studies continue to identify novel gene mutations in familial cases of ALS [315], many questions remain surrounding the pathological mechanisms associated with already established mutations, their roles in the disease phenotype, and the as-yet-undiscovered mechanisms that underly sporadic onset. The most investigated mechanisms revolve around neurocentric deficits in dysfunctional mitochondria and oxidative stress, axonal transport, glutamate excitotoxity, protein homeostasis, and RNA processing (Figure 4).

By detailing, as has been done in this review, the molecular events of the various pathways that are implicated in ALS, it becomes clear that these pathways can be linked to each other—in some cases, with one leading to another. For example, disrupted axonal transport can lead to an accumulation of nonfuctional mitochondria, while ATP deficiency and increased oxidative stress may damage proteins and DNA, which, in turn, could exacerbate the disruption of cellular homeostasis, leading to motor neuron death (Figure 4). These pathways are disrupted not only in motor neurons [24] but, also, in astrocytes [316,317], microglia [318,319], peripheral blood cells [43,320], and muscle [37,321,322,323], suggesting multisystemic [6] involvement in motor neuron death. Thus, by considering ALS from the perspective of shared molecular pathways [6,324], a cohesive understanding may yet emerge of the cellular mechanisms driving this pathology. It may be that different molecular pathways correspond to sub-strata of patients, such as among those with known genetic forms of ALS, as suggested in [6]. However, the identification of these strata may prove to be extremely challenging in non-monogenic forms of the disease [324].

## References

[1] Arthur K.C., Calvo A., Price T.R., Geiger J.T., Chiò A., Traynor B.J. (2016). Projected increase in amyotrophic lateral sclerosis from 2015 to 2040. Nat. Commun..

[2] Ragagnin A.M.G., Shadfar S., Vidal M., Jamali M.S., Atkin J.D. (2019). Motor Neuron Susceptibility in ALS/FTD. Front. Neurosci..

[3] Nicaise C., Mitrecic D., Pochet R. (2011). Brain and spinal cord affected by amyotrophic lateral sclerosis induce differential growth factors expression in rat mesenchymal and neural stem cells. Neuropathol. Appl. Neurobiol..

[4] Gibbons C., Pagnini F., Friede T., Young C.A. (2018). Treatment of fatigue in amyotrophic lateral sclerosis/motor neuron disease. Cochrane Database Syst. Rev..

[5] Turner M.R., Al-Chalabi A., Chio A., Hardiman O., Kiernan M.C., Rohrer J.D., Rowe J., Seeley W., Talbot K. (2017). Genetic screening in sporadic ALS and FTD. J. Neurol. Neurosurg. Psychiatry.

[6] Vijayakumar U.G., Milla V., Cynthia Stafford M.Y., Bjourson A.J., Duddy W., Duguez S.M.-R. (2019). A Systematic Review of Suggested Molecular Strata, Biomarkers and Their Tissue Sources in ALS. Front. Neurol..

[7] Connolly O., Le Gall L., McCluskey G., Donaghy C.G., Duddy W.J., Duguez S. (2020). A Systematic Review of Genotype–Phenotype Correlation across Cohorts Having Causal Mutations of Different Genes in ALS. J. Pers. Med..

[8] Watson M.R., Lagow R.D., Xu K., Zhang B., Bonini N.M. (2008). A drosophila model for amyotrophic lateral sclerosis reveals motor neuron damage by human SOD1. J. Biol. Chem..

[9] Estes P.S., Boehringer A., Zwick R., Tang J.E., Grigsby B., Zarnescu D.C. (2011). Wild-type and A315T mutant TDP-43 exert differential neurotoxicity in a Drosophila model of ALS. Hum. Mol. Genet..

[10] Chen Y., Yang M., Deng J., Chen X., Ye Y., Zhu L., Liu J., Ye H., Shen Y., Li Y. (2011). Expression of human FUS protein in Drosophila leads to progressive neurodegeneration. Protein Cell.

[11] Xu Z., Poidevin M., Li X., Li Y., Shu L., Nelson D.L., Li H., Hales C.M., Gearing M., Wingo T.S. (2013). Expanded GGGGCC repeat RNA associated with amyotrophic lateral sclerosis and frontotemporal dementia causes neurodegeneration. Proc. Natl. Acad. Sci. USA.

[12] Therrien M., Parker J.A. (2014). Worming forward: Amyotrophic lateral sclerosis toxicity mechanisms and genetic interactions in Caenorhabditis elegans. Front. Genet..

[13] Sakowski S.A., Lunn J.S., Busta A.S., Oh S.S., Zamora-Berridi G., Palmer M., Rosenberg A.A., Philip S.G., Dowling J.J., Feldman E.L. (2012). Neuromuscular effects of G93A-SOD1 expression in zebrafish. Mol. Neurodegener..

[14] Armstrong G.A.B., Drapeau P. (2013). Loss and gain of FUS function impair neuromuscular synaptic transmission in a genetic model of ALS. Hum. Mol. Genet..

[15] Ciura S., Lattante S., Le Ber I., Latouche M., Tostivint H., Brice A., Kabashi E. (2013). Loss of function of C9orf72 causes motor deficits in a zebrafish model of amyotrophic lateral sclerosis. Ann. Neurol..

[16] Schmid B., Hruscha A., Hogl S., Banzhaf-Strathmann J., Strecker K., van der Zee J., Teucke M., Eimer S., Hegermann J., Kittelmann M. (2013). Loss of ALS-associated TDP-43 in zebrafish causes muscle degeneration, vascular dysfunction, and reduced motor neuron axon outgrowth. Proc. Natl. Acad. Sci. USA.

[17] Gurney M.E., Pu H., Chiu A.Y., Dal Canto M.C., Polchow C.Y., Alexander D.D., Caliendo J., Hentati A., Kwon Y.W., Deng H.X. (1994). Motor neuron degeneration in mice that express a human Cu,Zn superoxide dismutase mutation. Science.

[18] Pansarasa O., Bordoni M., Drufuca L., Diamanti L., Sproviero D., Trotti R., Bernuzzi S., La Salvia S., Gagliardi S., Ceroni M. (2018). Lymphoblastoid cell lines as a model to understand amyotrophic lateral sclerosis disease mechanisms. Dis. Model. Mech..

[19] Boston-Howes W., Williams E.O., Bogush A., Scolere M., Pasinelli P., Trotti D. (2008). Nordihydroguaiaretic acid increases glutamate uptake in vitro and in vivo: Therapeutic implications for amyotrophic lateral sclerosis. Exp. Neurol..

[20] Moreno-Martet M., Mestre L., Loria F., Guaza C., Fernandez-Ruiz J., de Lago E. (2012). Identification of receptors and enzymes for endocannabinoids in NSC-34 cells: Relevance for in vitro studies with cannabinoids in motor neuron diseases. Neurosci. Lett..

[21] Van Damme P., Bogaert E., Dewil M., Hersmus N., Kiraly D., Scheveneels W., Bockx I., Braeken D., Verpoorten N., Verhoeven K. (2007). Astrocytes regulate GluR2 expression in motor neurons and their vulnerability to excitotoxicity. Proc. Natl. Acad. Sci. USA.

[22] Volk A.E., Weishaupt J.H., Andersen P.M., Ludolph A.C., Kubisch C. (2018). Current knowledge and recent insights into the genetic basis of amyotrophic lateral sclerosis. Med. Genet..

[23] Chia R., Chiò A., Traynor B.J. (2018). Novel genes associated with amyotrophic lateral sclerosis: Diagnostic and clinical implications. Lancet Neurol..

[24] Boillée S., Vande Velde C., Cleveland D.W. (2006). ALS: A Disease of Motor Neurons and Their Nonneuronal Neighbors. Neuron.

[25] Gupta R., Lan M., Mojsilovic-Petrovic J., Choi W.H., Safren N., Barmada S., Lee M.J., Kalb R. (2017). The Proline/Arginine Dipeptide from Hexanucleotide Repeat Expanded C9ORF72 Inhibits the Proteasome. Eneuro.

[26] Mitsumoto H., Santella R.M., Liu X., Bogdanov M., Zipprich J., Wu H.-C., Mahata J., Kilty M., Bednarz K., Bell D. (2008). Oxidative Stress Biomarkers in Sporadic ALS.

[27] Chang Y., Kong Q., Shan X., Tian G., Ilieva H., Cleveland D.W., Rothstein J.D., Borchelt D.R., Wong P.C., Lin C.G. (2008). Messenger RNA Oxidation Occurs Early in Disease Pathogenesis and Promotes Motor Neuron Degeneration in ALS. PLoS ONE.

[28] De Vos K.J., Chapman A.L., Tennant M.E., Manser C., Tudor E.L., Lau K.-F., Brownlees J., Ackerley S., Shaw P.J., Mcloughlin D.M. (2007). Familial amyotrophic lateral sclerosis-linked SOD1 mutants perturb fast axonal transport to reduce axonal mitochondria content Europe PMC Funders Group. Hum. Mol. Genet..

[29] Kiaei M., Kipiani K., Calingasan N., Wille E., Chen J., Heissig B., Rafii S., Lorenzl S., Beal M. (2007). Matrix metalloproteinase-9 regulates TNF-α and FasL expression in neuronal, glial cells and its absence extends life in a transgenic mouse model of amyotrophic lateral sclerosis. Exp. Neurol..

[30] Laslo P., Lipski J., Nicholson L.F.B., Miles G.B., Funk G.D. (2001). GluR2 AMPA Receptor Subunit Expression in Motoneurons at Low and High Risk for Degeneration in Amyotrophic Lateral Sclerosis. Exp. Neurol..

[31] Foerster B.R., Pomper M.G., Callaghan B.C., Petrou M., Edden R.A.E., Mohamed M.A., Welsh R.C., Carlos R.C., Barker P.B., Feldman E.L. (2013). An imbalance between excitatory and inhibitory neurotransmitters in amyotrophic lateral sclerosis revealed by use of 3-T proton magnetic resonance spectroscopy. JAMA Neurol..

[32] Kumar A., Bala L., Kalita J., Misra U.K., Singh R.L., Khetrapal C.L., Babu G.N. (2010). Metabolomic analysis of serum by (1) H NMR spectroscopy in amyotrophic lateral sclerosis. Clin. Chim. Acta.

[33] Spreux-Varoquaux O., Bensimon G., Lacomblez L., Salachas F., Pradat P.F., Le Forestier N., Marouan A., Dib M., Meininger V. (2002). Glutamate levels in cerebrospinal fluid in amyotrophic lateral sclerosis: A reappraisal using a new HPLC method with coulometric detection in a large cohort of patients. J. Neurol. Sci..

[34] Webster C.P., Smith E.F., Bauer C.S., Moller A., Hautbergue G.M., Ferraiuolo L., Myszczynska M.A., Higginbottom A., Walsh M.J., Whitworth A.J. (2016). The C9orf72 protein interacts with Rab1a and the ULK1 complex to regulate initiation of autophagy. EMBO J..

[35] Leigh P.N., Whitwell H., Garofalo O., Buller J., Swash M., Martin J.E., Gallo J.M., Weller R.O., Anderton B.H. (1991). Ubiquitin-immunoreactive intraneuronal inclusions in amyotrophic lateral sclerosis. Morphology, distribution, and specificity. Brain.

[36] Sreedharan J., Blair I.P., Tripathi V.B., Hu X., Vance C., Rogelj B., Ackerley S., Durnall J.C., Williams K.L., Buratti E. (2008). TDP-43 Mutations in Familial and Sporadic Amyotrophic Lateral Sclerosis. Science.

[37] Cykowski M.D., Dickson D.W., Powell S.Z., Arumanayagam A.S., Rivera A.L., Appel S.H. (2019). Dipeptide repeat (DPR) pathology in the skeletal muscle of ALS patients with C9ORF72 repeat expansion. Acta Neuropathol..

[38] Taylor J.P., Brown R.H., Cleveland D.W. (2016). Decoding ALS: From genes to mechanism. Nature.

[39] Lagier-Tourenne C., Polymenidou M., Hutt K.R., Vu A.Q., Baughn M., Huelga S.C., Clutario K.M., Ling S.-C., Liang T.Y., Mazur C. (2012). Divergent roles of ALS-linked proteins FUS/TLS and TDP-43 intersect in processing long pre-mRNAs. Nat. Neurosci..

[40] Haeusler A.R., Donnelly C.J., Rothstein J.D. (2016). The expanding biology of the C9orf72 nucleotide repeat expansion in neurodegenerative disease. Nat. Rev. Neurosci..

[41] Butti Z., Patten S.A. (2019). RNA Dysregulation in Amyotrophic Lateral Sclerosis. Front. Genet..

[42] Polymenidou M., Lagier-Tourenne C., Hutt K.R., Huelga S.C., Moran J., Liang T.Y., Ling S.-C., Sun E., Wancewicz E., Mazur C. (2011). Long pre-mRNA depletion and RNA missplicing contribute to neuronal vulnerability from loss of TDP-43. Nat. Neurosci..

[43] Rizzo F., Riboldi G., Salani S., Nizzardo M., Simone C., Corti S., Hedlund E. (2014). Cellular therapy to target neuroinflammation in amyotrophic lateral sclerosis. Cell. Mol. Life Sci..

[44] Dobrowolny G., Aucello M., Rizzuto E., Beccafico S., Mammucari C., Boncompagni S., Bonconpagni S., Belia S., Wannenes F., Nicoletti C. (2008). Skeletal muscle is a primary target of SOD1G93A-mediated toxicity. Cell Metab..

[45] Betteridge D.J. (2000). What is oxidative stress?. Metabolism.

[46] Kandlur A., Satyamoorthy K., Gangadharan G. (2020). Oxidative Stress in Cognitive and Epigenetic Aging: A Retrospective Glance. Front. Mol. Neurosci..

[47] Shaw P.J., Ince P.G., Falkous G., Mantle D. (1995). Oxidative damage to protein in sporadic motor neuron disease spinal cord. Ann. Neurol..

[48] Rosen D.R. (1993). Mutations in Cu/Zn superoxide dismutase gene are associated with familial amyotrophic lateral sclerosis. Nature.

[49] Zarei S., Carr K., Reiley L., Diaz K., Guerra O., Altamirano P.F., Pagani W., Lodin D., Orozco G., Chinea A. (2015). A Comprehensive Review of Amyotrophic Lateral Sclerosis.

[50] Shibata N., Nagai R., Uchida K., Horiuchi S., Yamada S., Hirano A., Kawaguchi M., Yamamoto T., Sasaki S., Kobayashi M. (2001). Morphological evidence for lipid peroxidation and protein glycoxidation in spinal cords from sporadic amyotrophic lateral sclerosis patients. Brain Res..

[51] Juarez J.C., Manuia M., Burnett M.E., Betancourt O., Boivin B., Shaw D.E., Tonks N.K., Mazar A.P., Donate F. (2008). Superoxide dismutase 1 (SOD1) is essential for H2O2-mediated oxidation and inactivation of phosphatases in growth factor signaling. Proc. Natl. Acad. Sci. USA.

[52] Crosby K., Crown A.M., Roberts B.L., Brown H., Ayers J.I., Borchelt D.R. (2018). Loss of charge mutations in solvent exposed Lys residues of superoxide dismutase 1 do not induce inclusion formation in cultured cell models. PLoS ONE.

[53] Saccon R.A., Bunton-Stasyshyn R.K.A., Fisher E.M.C., Fratta P. (2013). Is SOD1 loss of function involved in amyotrophic lateral sclerosis?. Brain.

[54] Kirby J., Halligan E., Baptista M.J., Allen S., Heath P.R., Holden H., Barber S.C., Loynes C.A., Wood-Allum C.A., Lunec J. (2005). Mutant SOD1 alters the motor neuronal transcriptome: Implications for familial ALS. Brain.

[55] Johnson J.A., Johnson D.A., Kraft A.D., Calkins M.J., Jakel R.J., Vargas M.R., Chen P.-C. (2008). The Nrf2-ARE Pathway. Ann. N. Y. Acad. Sci..

[56] Shibata N., Hirano A., Kato S., Nagai R., Horiuchi S., Komori T., Umahara T., Asayama K., Kobayashi M. (1999). Advanced glycation endproducts are deposited in neuronal hyaline inclusions: A study on familial amyotrophic lateral sclerosis with superoxide dismutase-1 mutation. Acta Neuropathol..

[57] Andrus P.K., Fleck T.J., Gurney M.E., Hall E.D. (2002). Protein Oxidative Damage in a Transgenic Mouse Model of Familial Amyotrophic Lateral Sclerosis. J. Neurochem..

[58] Hall E.D., Andrus P.K., Oostveen J.A., Fleck T.J., Gurney M.E. (1998). Relationship of oxygen radical-induced lipid peroxidative damage to disease onset and progression in a transgenic model of familial ALS. J. Neurosci. Res..

[59] Elden A.C., Kim H.-J., Hart M.P., Chen-Plotkin A.S., Johnson B.S., Fang X., Armakola M., Geser F., Greene R., Lu M.M. (2010). Ataxin-2 intermediate-length polyglutamine expansions are associated with increased risk for ALS. Nature.

[60] van Blitterswijk M., Mullen B., Heckman M.G., Baker M.C., DeJesus-Hernandez M., Brown P.H., Murray M.E., Hsiung G.Y.R., Stewart H., Karydas A.M. (2014). Ataxin-2 as potential disease modifier in C9ORF72 expansion carriers. Neurobiol. Aging.

[61] Chiò A., Calvo A., Moglia C., Canosa A., Brunetti M., Barberis M., Restagno G., Conte A., Bisogni G., Marangi G. (2015). ATXN2 polyQ intermediate repeats are a modifier of ALS survival. Neurology.

[62] Bertoni A., Giuliano P., Galgani M., Rotoli D., Ulianich L., Adornetto A., Santillo M.R., Porcellini A., Avvedimento V.E. (2011). Early and Late Events Induced by PolyQ-expanded Proteins. J. Biol. Chem..

[63] Polci R., Peng A., Chen P.-L., Riley D.J., Chen Y. (2004). NIMA-Related Protein Kinase 1 Is Involved Early in the Ionizing Radiation-Induced DNA Damage Response. Cancer Res..

[64] Fang X., Lin H., Wang X., Zuo Q., Qin J., Zhang P. (2015). The NEK1 interactor, C21ORF2, is required for efficient DNA damage repair. Acta Biochim. Biophys. Sin. (Shanghai).

[65] Kannan A., Bhatia K., Branzei D., Gangwani L. (2018). Combined deficiency of Senataxin and DNA-PKcs causes DNA damage accumulation and neurodegeneration in spinal muscular atrophy. Nucleic Acids Res..

[66] Cirulli E.T., Lasseigne B.N., Petrovski S., Sapp P.C., Dion P.A., Leblond C.S., Couthouis J., Lu Y.-F., Wang Q., Krueger B.J. (2015). Exome sequencing in amyotrophic lateral sclerosis identifies risk genes and pathways. Science.

[67] van Rheenen W., Shatunov A., Dekker A.M., McLaughlin R.L., Diekstra F.P., Pulit S.L., van der Spek R.A.A., Võsa U., de Jong S., Robinson M.R. (2016). Genome-wide association analyses identify new risk variants and the genetic architecture of amyotrophic lateral sclerosis. Nat. Genet..

[68] Chen Y.-Z., Bennett C.L., Huynh H.M., Blair I.P., Puls I., Irobi J., Dierick I., Abel A., Kennerson M.L., Rabin B.A. (2004). DNA/RNA helicase gene mutations in a form of juvenile amyotrophic lateral sclerosis (ALS4). Am. J. Hum. Genet..

[69] Kenna K.P., Van Doormaal P.T.C., Dekker A.M., Ticozzi N., Kenna B.J., Diekstra F.P., Van Rheenen W., Van Eijk K.R., Jones A.R., Keagle P. (2016). NEK1 variants confer susceptibility to amyotrophic lateral sclerosis. Nat. Genet..

[70] Higelin J., Catanese A., Semelink-Sedlacek L.L., Oeztuerk S., Lutz A.K., Bausinger J., Barbi G., Speit G., Andersen P.M., Ludolph A.C. (2018). NEK1 loss-of-function mutation induces DNA damage accumulation in ALS patient-derived motoneurons. Stem Cell Res..

[71] Bennett C.L., Dastidar S.G., Ling S.C., Malik B., Ashe T., Wadhwa M., Miller D.B., Lee C., Mitchell M.B., van Es M.A. (2018). Senataxin mutations elicit motor neuron degeneration phenotypes and yield TDP-43 mislocalization in ALS4 mice and human patients. Acta Neuropathol..

[72] Kowaltowski A.J., Vercesi A.E. (1999). Mitochondrial damage induced by conditions of oxidative stress. Free Radic. Biol. Med..

[73] Malhotra J.D., Kaufman R.J. (2007). Endoplasmic Reticulum Stress and Oxidative Stress: A Vicious Cycle or a Double-Edged Sword?. Antioxid. Redox Signal..

[74] Reichmann D., Voth W., Jakob U. (2018). Maintaining a Healthy Proteome during Oxidative Stress. Mol. Cell.

[75] McBride H.M., Neuspiel M., Wasiak S. (2006). Mitochondria: More Than Just a Powerhouse. Curr. Biol..

[76] Sasaki S., Horie Y., Iwata M. (2007). Mitochondrial alterations in dorsal root ganglion cells in sporadic amyotrophic lateral sclerosis. Acta Neuropathol..

[77] Cogliati S., Frezza C., Soriano M.E., Varanita T., Quintana-Cabrera R., Corrado M., Cipolat S., Costa V., Casarin A., Gomes L.C. (2013). Mitochondrial Cristae Shape Determines Respiratory Chain Supercomplexes Assembly and Respiratory Efficiency. Cell.

[78] Zerbes R.M., Bohnert M., Stroud D.A., von der Malsburg K., Kram A., Oeljeklaus S., Warscheid B., Becker T., Wiedemann N., Veenhuis M. (2012). Role of MINOS in Mitochondrial Membrane Architecture: Cristae Morphology and Outer Membrane Interactions Differentially Depend on Mitofilin Domains. J. Mol. Biol..

[79] Pánek T., Eliáš M., Vancová M., Lukeš J., Hashimi H. (2020). Returning to the Fold for Lessons in Mitochondrial Crista Diversity and Evolution. Curr. Biol..

[80] Genin E.C., Plutino M., Bannwarth S., Villa E., Cisneros-Barroso E., Roy M., Ortega-Vila B., Fragaki K., Lespinasse F., Pinero-Martos E. (2016). CHCHD10 mutations promote loss of mitochondrial cristae junctions with impaired mitochondrial genome maintenance and inhibition of apoptosis. EMBO Mol. Med..

[81] Genin E.C., Bannwarth S., Lespinasse F., Ortega-Vila B., Fragaki K., Itoh K., Villa E., Lacas-Gervais S., Jokela M., Auranen M. (2018). Loss of MICOS complex integrity and mitochondrial damage, but not TDP-43 mitochondrial localisation, are likely associated with severity of CHCHD10-related diseases. Neurobiol. Dis..

[82] Zhou W., Ma D., Sun A.X., Tran H.-D., Ma D., Singh B.K., Zhou J., Zhang J., Wang D., Zhao Y. (2019). PD-linked CHCHD2 mutations impair CHCHD10 and MICOS complex leading to mitochondria dysfunction. Hum. Mol. Genet..

[83] Purandare N., Somayajulu M., Hüttemann M., Grossman L.I., Aras S. (2018). The cellular stress proteins CHCHD10 and MNRR1 (CHCHD2): Partners in mitochondrial and nuclear function and dysfunction. J. Biol. Chem..

[84] Deng J., Yang M., Chen Y., Chen X., Liu J., Sun S., Cheng H., Li Y., Bigio E.H., Mesulam M. (2015). FUS Interacts with HSP60 to Promote Mitochondrial Damage. PLoS Genet..

[85] Deng J., Wang P., Chen X., Cheng H., Liu J., Fushimi K., Zhu L., Wu J.Y. (2018). FUS interacts with ATP synthase beta subunit and induces mitochondrial unfolded protein response in cellular and animal models. Proc. Natl. Acad. Sci. USA.

[86] Crozat A., Aman P., Mandahl N., Ron D. (1993). Fusion of CHOP to a novel RNA-binding protein in human myxoid liposarcoma. Nature.

[87] Vance C., Rogelj B., Hortobagyi T., De Vos K.J., Nishimura A.L., Sreedharan J., Hu X., Smith B., Ruddy D., Wright P. (2009). Mutations in FUS, an RNA Processing Protein, Cause Familial Amyotrophic Lateral Sclerosis Type 6. Science.

[88] Kwiatkowski T.J., Bosco D.A., LeClerc A.L., Tamrazian E., Vanderburg C.R., Russ C., Davis A., Gilchrist J., Kasarskis E.J., Munsat T. (2009). Mutations in the FUS/TLS Gene on Chromosome 16 Cause Familial Amyotrophic Lateral Sclerosis. Science.

[89] Hardiman O., Al-Chalabi A., Chio A., Corr E.M., Logroscino G., Robberecht W., Shaw P.J., Simmons Z., van den Berg L.H. (2017). Amyotrophic lateral sclerosis. Nat. Rev. Dis. Prim..

[90] Magrané J., Cortez C., Gan W.-B., Manfredi G. (2014). Abnormal mitochondrial transport and morphology are common pathological denominators in SOD1 and TDP43 ALS mouse models. Hum. Mol. Genet..

[91] Bernardini C., Censi F., Lattanzi W., Barba M., Calcagnini G., Giuliani A., Tasca G., Sabatelli M., Ricci E., Michetti F. (2013). Mitochondrial network genes in the skeletal muscle of amyotrophic lateral sclerosis patients. PLoS ONE.

[92] Higgins C.M.J., Jung C., Xu Z. (2003). ALS-associated mutant SOD1G93A causes mitochondrial vacuolation by expansion of the intermembrane space and by involvement of SOD1 aggregation and peroxisomes. BMC Neurosci..

[93] Pasinelli P., Belford M.E., Lennon N., Bacskai B.J., Hyman B.T., Trotti D., Brown R.H. (2004). Amyotrophic Lateral Sclerosis-Associated SOD1 Mutant Proteins Bind and Aggregate with Bcl-2 in Spinal Cord Mitochondria. Neuron.

[94] Kausar S., Wang F., Cui H. (2018). The Role of Mitochondria in Reactive Oxygen Species Generation and Its Implications for Neurodegenerative Diseases. Cells.

[95] Browne S.E., Bowling A.C., Baik M.J., Gurney M., Brown R.H., Beal M.F. (2002). Metabolic Dysfunction in Familial, but Not Sporadic, Amyotrophic Lateral Sclerosis. J. Neurochem..

[96] Wiedemann F.R., Manfredi G., Mawrin C., Beal M.F., Schon E.A. (2002). Mitochondrial DNA and respiratory chain function in spinal cords of ALS patients. J. Neurochem..

[97] Dröse S., Brandt U. (2012). Molecular Mechanisms of Superoxide Production by the Mitochondrial Respiratory Chain. Mitochondrial Oxidative Phosphorylation.

[98] Umoh M.E., Fournier C., Li Y., Polak M., Shaw L., Landers J.E., Hu W., Gearing M., Glass J.D. (2016). Comparative analysis of C9orf72 and sporadic disease in an ALS clinic population. Neurology.

[99] DeJesus-Hernandez M., Mackenzie I.R.R., Boeve B.F.F., Boxer A.L.L., Baker M., Rutherford N.J.J., Nicholson A.M.M., Finch N.A.A., Flynn H., Adamson J. (2011). Expanded GGGGCC hexanucleotide repeat in noncoding region of C9ORF72 causes chromosome 9p-linked FTD and ALS. Neuron.

[100] Renton A.E., Majounie E., Waite A., Simón-Sánchez J., Rollinson S., Gibbs J.R., Schymick J.C., Laaksovirta H., van Swieten J.C., Myllykangas L. (2011). A Hexanucleotide Repeat Expansion in C9ORF72 Is the Cause of Chromosome 9p21-Linked ALS-FTD. Neuron.

[101] Balendra R., Isaacs A.M. (2018). C9orf72-mediated ALS and FTD: Multiple pathways to disease. Nat. Rev. Neurol..

[102] Choi S.Y., Lopez-Gonzalez R., Krishnan G., Phillips H.L., Li A.N., Seeley W.W., Yao W.-D., Almeida S., Gao F.-B. (2019). C9ORF72-ALS/FTD-associated poly(GR) binds Atp5a1 and compromises mitochondrial function in vivo. Nat. Neurosci..

[103] Onesto E., Colombrita C., Gumina V., Borghi M.O., Dusi S., Doretti A., Fagiolari G., Invernizzi F., Moggio M., Tiranti V. (2016). Gene-specific mitochondria dysfunctions in human TARDBP and C9ORF72 fibroblasts. Acta Neuropathol. Commun..

[104] Birger A., Ben-Dor I., Ottolenghi M., Turetsky T., Gil Y., Sweetat S., Perez L., Belzer V., Casden N., Steiner D. (2019). Human iPSC-derived astrocytes from ALS patients with mutated C9ORF72 show increased oxidative stress and neurotoxicity. EBioMedicine.

[105] Youle R.J., Narendra D.P. (2011). Mechanisms of mitophagy. Nat. Rev. Mol. Cell Biol..

[106] Moore A.S., Holzbaur E.L.F. (2016). Dynamic recruitment and activation of ALS-associated TBK1 with its target optineurin are required for efficient mitophagy. Proc. Natl. Acad. Sci. USA.

[107] Maruyama H., Morino H., Ito H., Izumi Y., Kato H., Watanabe Y., Kinoshita Y., Kamada M., Nodera H., Suzuki H. (2010). Mutations of optineurin in amyotrophic lateral sclerosis. Nature.

[108] Fivenson E.M., Lautrup S., Sun N., Scheibye-Knudsen M., Stevnsner T., Nilsen H., Bohr V.A., Fang E.F. (2017). Mitophagy in neurodegeneration and aging. Neurochem. Int..

[109] Damiano M., Starkov A.A., Petri S., Kipiani K., Kiaei M., Mattiazzi M., Flint Beal M., Manfredi G. (2006). Neural mitochondrial Ca^2+^ capacity impairment precedes the onset of motor symptoms in G93A Cu/Zn-superoxide dismutase mutant mice. J. Neurochem..

[110] Sathasivam S., Grierson A.J., Shaw P.J., Shaw P.J. (2005). Characterization of the caspase cascade in a cell culture model of SOD1-related familial amyotrophic lateral sclerosis: Expression, activation and therapeutic effects of inhibition. Neuropathol. Appl. Neurobiol..

[111] Sutton M.A., Schuman E.M. (2006). Dendritic Protein Synthesis, Synaptic Plasticity, and Memory. Cell.

[112] Yuan A., Rao M.V., Veeranna, Nixon R.A. (2017). Neurofilaments and Neurofilament Proteins in Health and Disease. Cold Spring Harb. Perspect. Biol..

[113] Brady S. (1995). Mice overexpressing the human neurofilament heavy gene as a model of ALS. Neurobiol. Aging.

[114] Prokop A. (2020). Cytoskeletal organization of axons in vertebrates and invertebrates. J. Cell Biol..

[115] Figlewicz D.A., Krizus A., Martinoli M.G., Meininger V., Dib M., Rouleau G.A., Julien J.P. (1994). Variants of the heavy neurofilament subunit are associated with the development of amyotrophic lateral sclerosis. Hum. Mol. Genet..

[116] Dubey S., Bhembre N., Bodas S., Veer S., Ghose A., Callan-Jones A., Pullarkat P. (2020). The axonal actin-spectrin lattice acts as a tension buffering shock absorber. Elife.

[117] Witke W., Podtelejnikov A.V., Di Nardo A., Sutherland J.D., Gurniak C.B., Dotti C., Mann M. (1998). In mouse brain profilin I and profilin II associate with regulators of the endocytic pathway and actin assembly. EMBO J..

[118] Wu C.-H., Fallini C., Ticozzi N., Keagle P.J., Sapp P.C., Piotrowska K., Lowe P., Koppers M., McKenna-Yasek D., Baron D.M. (2012). Mutations in the profilin 1 gene cause familial amyotrophic lateral sclerosis. Nature.

[119] Fil D., DeLoach A., Yadav S., Alkam D., MacNicol M., Singh A., Compadre C.M., Goellner J.J., O’Brien C.A., Fahmi T. (2017). Mutant Profilin1 transgenic mice recapitulate cardinal features of motor neuron disease. Hum. Mol. Genet..

[120] Tempes A., Weslawski J., Brzozowska A., Jaworski J. (2020). Role of dynein-dynactin complex, kinesins, motor adaptors, and their phosphorylation in dendritogenesis. J. Neurochem..

[121] Uchida A., Alami N.H., Brown A. (2009). Tight Functional Coupling of Kinesin-1A and Dynein Motors in the Bidirectional Transport of Neurofilaments. Mol. Biol. Cell.

[122] Heisler F.F., Lee H.K., Gromova K.V., Pechmann Y., Schurek B., Ruschkies L., Schroeder M., Schweizer M., Kneussel M. (2014). GRIP1 interlinks N-cadherin and AMPA receptors at vesicles to promote combined cargo transport into dendrites. Proc. Natl. Acad. Sci. USA.

[123] Nakajima K., Yin X., Takei Y., Seog D.-H., Homma N., Hirokawa N. (2012). Molecular motor KIF5A is essential for GABA(A) receptor transport, and KIF5A deletion causes epilepsy. Neuron.

[124] Smith B.N., Ticozzi N., Fallini C., Gkazi A.S., Topp S., Kenna K.P., Scotter E.L., Kost J., Keagle P., Miller J.W. (2014). Exome-wide Rare Variant Analysis Identifies TUBA4A Mutations Associated with Familial ALS. Neuron.

[125] Puls I., Jonnakuty C., LaMonte B.H., Holzbaur E.L.F., Tokito M., Mann E., Floeter M.K., Bidus K., Drayna D., Oh S.J. (2003). Mutant dynactin in motor neuron disease. Nat. Genet..

[126] LaMonte B.H., Wallace K.E., Holloway B.A., Shelly S.S., Ascano J., Tokito M., Van Winkle T., Howland D.S., Holzbaur E.L.F. (2002). Disruption of dynein/dynactin inhibits axonal transport in motor neurons causing late-onset progressive degeneration. Neuron.

[127] Miki H., Setou M., Kaneshiro K., Hirokawa N. (2001). All kinesin superfamily protein, KIF, genes in mouse and human. Proc. Natl. Acad. Sci. USA.

[128] Nicolas A., Kenna K.P., Renton A.E., Ticozzi N., Faghri F., Chia R., Dominov J.A., Kenna B.J., Nalls M.A., Keagle P. (2018). Genome-wide Analyses Identify KIF5A as a Novel ALS Gene. Neuron.

[129] Karle K.N., Möckel D., Reid E., Schöls L. (2012). Axonal transport deficit in a KIF5A(-/-) mouse model. Neurogenetics.

[130] Warita H., Itoyama Y., Abe K. (1999). Selective impairment of fast anterograde axonal transport in the peripheral nerves of asymptomatic transgenic mice with a G93A mutant SOD1 gene. Brain Res..

[131] Fischer L.R., Culver D.G., Tennant P., Davis A.A., Wang M., Castellano-Sanchez A., Khan J., Polak M.A., Glass J.D. (2004). Amyotrophic lateral sclerosis is a distal axonopathy: Evidence in mice and man. Exp. Neurol..

[132] Tallon C., Russell K.A., Sakhalkar S., Andrapallayal N., Farah M.H. (2016). Length-dependent axo-terminal degeneration at the neuromuscular synapses of type II muscle in SOD1 mice. Neuroscience.

[133] Pantelidou M., Zographos S.E., Lederer C.W., Kyriakides T., Pfaffl M.W., Santama N. (2007). Differential expression of molecular motors in the motor cortex of sporadic ALS. Neurobiol. Dis..

[134] Landers J.E., Melki J., Meininger V., Glass J.D., van den Berg L.H., van Es M.A., Sapp P.C., van Vught P.W.J., McKenna-Yasek D.M., Blauw H.M. (2009). Reduced expression of the Kinesin-Associated Protein 3 (KIFAP3) gene increases survival in sporadic amyotrophic lateral sclerosis. Proc. Natl. Acad. Sci. USA.

[135] Ferraiuolo L., Kirby J., Grierson A.J., Sendtner M., Shaw P.J. (2011). Molecular pathways of motor neuron injury in amyotrophic lateral sclerosis. Nat. Rev. Neurol..

[136] Pitt D., Werner P., Raine C.S. (2000). Glutamate excitotoxicity in a model of multiple sclerosis. Nat. Med..

[137] Vaarmann A., Kovac S., Holmström K.M., Gandhi S., Abramov A.Y. (2013). Dopamine protects neurons against glutamate-induced excitotoxicity. Cell Death Dis..

[138] Rothstein J.D., Tsai G., Kuncl R.W., Clawson L., Cornblath D.R., Drachman D.B., Pestronk A., Stauch B.L., Coyle J.T. (1990). Abnormal excitatory amino acid metabolism in amyotrophic lateral sclerosis. Ann. Neurol..

[139] Shaw P.J., Forrest V., Ince P.G., Richardson J.P., Wastell H.J. (1995). CSF and Plasma Amino Acid Levels in Motor Neuron Disease: Elevation of CSF Glutamate in a Subset of Patients. Neurodegeneration.

[140] Wang Y., Qin Z. (2010). Molecular and cellular mechanisms of excitotoxic neuronal death. Apoptosis.

[141] Netzahualcoyotzi C., Tapia R. (2015). Degeneration of spinal motor neurons by chronic AMPA-induced excitotoxicity in vivo and protection by energy substrates. Acta Neuropathol. Commun..

[142] Van Den Bosch L., Vandenberghe W., Klaassen H., Van Houtte E., Robberecht W. (2000). Ca2+-permeable AMPA receptors and selective vulnerability of motor neurons. J. Neurol. Sci..

[143] Gregory J.M., Livesey M.R., McDade K., Selvaraj B.T., Barton S.K., Chandran S., Smith C. (2020). Dysregulation of AMPA receptor subunit expression in sporadic ALS post-mortem brain. J. Pathol..

[144] Traynelis S.F., Wollmuth L.P., McBain C.J., Menniti F.S., Vance K.M., Ogden K.K., Hansen K.B., Yuan H., Myers S.J., Dingledine R. (2010). Glutamate Receptor Ion Channels: Structure, Regulation, and Function. Pharmacol. Rev..

[145] Corona J.C., Tapia R. (2004). AMPA receptor activation, but not the accumulation of endogenous extracellular glutamate, induces paralysis and motor neuron death in rat spinal cord in vivo. J. Neurochem..

[146] Konen L.M., Wright A.L., Royle G.A., Morris G.P., Lau B.K., Seow P.W., Zinn R., Milham L.T., Vaughan C.W., Vissel B. (2020). A new mouse line with reduced GluA2 Q/R site RNA editing exhibits loss of dendritic spines, hippocampal CA1-neuron loss, learning and memory impairments and NMDA receptor-independent seizure vulnerability. Mol. Brain.

[147] Heath P.R., Tomkins J., Ince P.G., Shaw P.J. (2002). Quantitative assessment of AMPA receptor mRNA in human spinal motor neurons isolated by laser capture microdissection. Neuroreport.

[148] Takuma H., Kwak S., Yoshizawa T., Kanazawa I. (1999). Reduction of GluR2 RNA editing, a molecular change that increases calcium influx through AMPA receptors, selective in the spinal ventral gray of patients with amyotrophic lateral sclerosis. Ann. Neurol..

[149] Ince P., Stout N., Shaw P., Slade J., Hunziker W., Heizmann C.W., Baimbridge K.G. (1993). Parvalbumin and calbindin D-28k in the human motor system and in motor neuron disease. Neuropathol. Appl. Neurobiol..

[150] Higuchi M., Maas S., Single F.N., Hartner J., Rozov A., Burnashev N., Feldmeyer D., Sprengel R., Seeburg P.H. (2000). Point mutation in an AMPA receptor gene rescues lethality in mice deficient in the RNA-editing enzyme ADAR2. Nature.

[151] Hideyama T., Kwak S. (2011). When Does ALS Start? ADAR2?GluA2 Hypothesis for the Etiology of Sporadic ALS. Front. Mol. Neurosci..

[152] Aizawa H., Sawada J., Hideyama T., Yamashita T., Katayama T., Hasebe N., Kimura T., Yahara O., Kwak S. (2010). TDP-43 pathology in sporadic ALS occurs in motor neurons lacking the RNA editing enzyme ADAR2. Acta Neuropathol..

[153] Medina L., Figueredo-Cardenas G., Rothstein J.D., Reiner A. (1996). Differential Abundance of Glutamate Transporter Subtypes in Amyotrophic Lateral Sclerosis (ALS)-Vulnerable versus ALS-Resistant Brain Stem Motor Cell Groups. Exp. Neurol..

[154] Rothstein J.D., Dykes-Hoberg M., Pardo C.A., Bristol L.A., Jin L., Kuncl R.W., Kanai Y., Hediger M.A., Wang Y., Schielke J.P. (1996). Knockout of glutamate transporters reveals a major role for astroglial transport in excitotoxicity and clearance of glutamate. Neuron.

[155] Kong Q., Chang L.-C., Takahashi K., Liu Q., Schulte D.A., Lai L., Ibabao B., Lin Y., Stouffer N., Mukhopadhyay C.D. (2014). Small-molecule activator of glutamate transporter EAAT2 translation provides neuroprotection. J. Clin. Investig..

[156] Rothstein J.D., Patel S., Regan M.R., Haenggeli C., Huang Y.H., Bergles D.E., Jin L., Dykes Hoberg M., Vidensky S., Chung D.S. (2005). β-Lactam antibiotics offer neuroprotection by increasing glutamate transporter expression. Nature.

[157] Bristol L.A., Rothstein J.D. (1996). Glutamate transporter gene expression in amyotrophic lateral sclerosis motor cortex. Ann. Neurol..

[158] Sasaki S., Komori T., Iwata M. (2000). Excitatory amino acid transporter 1 and 2 immunoreactivity in the spinal cord in amyotrophic lateral sclerosis. Acta Neuropathol..

[159] Milanese M., Zappettini S., Onofri F., Musazzi L., Tardito D., Bonifacino T., Messa M., Racagni G., Usai C., Benfenati F. (2011). Abnormal exocytotic release of glutamate in a mouse model of amyotrophic lateral sclerosis. J. Neurochem..

[160] van Zundert B., Peuscher M.H., Hynynen M., Chen A., Neve R.L., Brown R.H., Constantine-Paton M., Bellingham M.C. (2008). Neonatal Neuronal Circuitry Shows Hyperexcitable Disturbance in a Mouse Model of the Adult-Onset Neurodegenerative Disease Amyotrophic Lateral Sclerosis. J. Neurosci..

[161] Rothstein J.D., Martin L.J., Kuncl R.W. (1992). Decreased Glutamate Transport by the Brain and Spinal Cord in Amyotrophic Lateral Sclerosis. N. Engl. J. Med..

[162] Vucic S., Nicholson G.A., Kiernan M.C. (2008). Cortical hyperexcitability may precede the onset of familial amyotrophic lateral sclerosis. Brain.

[163] Perry T.L., Krieger C., Hansen S., Eisen A. (1990). Amyotrophic lateral sclerosis: Amino acid levels in plasma and cerebrospinal fluid. Ann. Neurol..

[164] Perry T.L., Hansen S., Jones K. (1987). Brain glutamate deficiency in amyotrophic lateral sclerosis. Neurology.

[165] Wuolikainen A., Moritz T., Marklund S.L., Antti H., Andersen P.M. (2011). Disease-related changes in the cerebrospinal fluid metabolome in amyotrophic lateral sclerosis detected by GC/TOFMS. PLoS ONE.

[166] Shi Y., Hung S.-T., Rocha G., Lin S., Linares G.R., Staats K.A., Seah C., Wang Y., Chickering M., Lai J. (2019). Identification and therapeutic rescue of autophagosome and glutamate receptor defects in C9ORF72 and sporadic ALS neurons. JCI Insight.

[167] Selvaraj B.T., Livesey M.R., Zhao C., Gregory J.M., James O.T., Cleary E.M., Chouhan A.K., Gane A.B., Perkins E.M., Dando O. (2018). C9ORF72 repeat expansion causes vulnerability of motor neurons to Ca2+-permeable AMPA receptor-mediated excitotoxicity. Nat. Commun..

[168] Shi Y., Lin S., Staats K.A., Li Y., Chang W.-H., Hung S.-T., Hendricks E., Linares G.R., Wang Y., Son E.Y. (2018). Haploinsufficiency leads to neurodegeneration in C9ORF72 ALS/FTD human induced motor neurons. Nat. Med..

[169] Xiao S., McKeever P.M., Lau A., Robertson J. (2019). Synaptic localization of C9orf72 regulates post-synaptic glutamate receptor 1 levels. Acta Neuropathol. Commun..

[170] Tang D., Sheng J., Xu L., Zhan X., Liu J., Jiang H., Shu X., Liu X., Zhang T., Jiang L. (2020). Cryo-EM structure of C9ORF72–SMCR8–WDR41 reveals the role as a GAP for Rab8a and Rab11a. Proc. Natl. Acad. Sci. USA.

[171] Lai C., Xie C., Shim H., Chandran J., Howell B.W., Cai H. (2009). Regulation of endosomal motility and degradation by amyotrophic lateral sclerosis 2/alsin. Mol. Brain.

[172] Blasco H., Mavel S., Corcia P., Gordon P.H. (2014). The glutamate hypothesis in ALS: Pathophysiology and drug development. Curr. Med. Chem..

[173] Petrov D., Mansfield C., Moussy A., Hermine O. (2017). ALS clinical trials review: 20 years of failure. Are we any closer to registering a new treatment?. Front. Aging Neurosci..

[174] Thorley M., Malatras A., Duddy W.J., Le Gall L., Mouly V., Butler Browne G., Duguez S.M.-R. (2015). Changes in communication between muscle stem cells and their environment with aging. J. Neuromuscul. Dis..

[175] Paolicelli R.C., Bergamini G., Rajendran L. (2019). Cell-to-cell Communication by Extracellular Vesicles: Focus on Microglia. Neuroscience.

[176] Théry C. (2011). Exosomes: Secreted vesicles and intercellular communications. F1000 Biol. Rep..

[177] Raposo G., Stoorvogel W. (2013). Extracellular vesicles: Exosomes, microvesicles, and friends. J. Cell Biol..

[178] Basso M., Pozzi S., Tortarolo M., Fiordaliso F., Bisighini C., Pasetto L., Spaltro G., Lidonnici D., Gensano F., Battaglia E. (2013). Mutant copper-zinc superoxide dismutase (SOD1) induces protein secretion pathway alterations and exosome release in astrocytes: Implications for disease spreading and motor neuron pathology in amyotrophic lateral sclerosis. J. Biol. Chem..

[179] Silverman J.M., Christy D., Shyu C.C., Moon K.-M., Fernando S., Gidden Z., Cowan C.M., Ban Y., Stacey R.G., Grad L.I. (2019). CNS-derived extracellular vesicles from superoxide dismutase 1 (SOD1)G93A ALS mice originate from astrocytes and neurons and carry misfolded SOD1. J. Biol. Chem..

[180] Sproviero D., La Salvia S., Colombo F., Zucca S., Pansarasa O., Diamanti L., Costa A., Lova L., Giannini M., Gagliardi S. (2019). Leukocyte Derived Microvesicles as Disease Progression Biomarkers in Slow Progressing Amyotrophic Lateral Sclerosis Patients. Front. Neurosci..

[181] Chen Y., Xia K., Chen L., Fan D. (2019). Increased Interleukin-6 Levels in the Astrocyte-Derived Exosomes of Sporadic Amyotrophic Lateral Sclerosis Patients. Front. Neurosci..

[182] Westergard T., Jensen B.K., Wen X., Cai J., Kropf E., Iacovitti L., Pasinelli P., Trotti D. (2016). Cell-to-Cell Transmission of Dipeptide Repeat Proteins Linked to C9orf72 -ALS/FTD. Cell Rep..

[183] Scaramozza A., Marchese V., Papa V., Salaroli R., Sorarù G., Angelini C., Cenacchi G. (2014). Skeletal Muscle Satellite Cells in Amyotrophic Lateral Sclerosis. Ultrastruct. Pathol..

[184] Parkinson N., Ince P.G., Smith M.O., Highley R., Skibinski G., Andersen P.M., Morrison K.E., Pall H.S., Hardiman O., Collinge J. (2006). ALS phenotypes with mutations in CHMP2B (charged multivesicular body protein 2B). Neurology.

[185] Skibinski G., Parkinson N.J., Brown J.M., Chakrabarti L., Lloyd S.L., Hummerich H., Nielsen J.E., Hodges J.R., Spillantini M.G., Thusgaard T. (2005). Mutations in the endosomal ESCRTIII-complex subunit CHMP2B in frontotemporal dementia. Nat. Genet..

[186] Blanc L., Vidal M. (2018). New insights into the function of Rab GTPases in the context of exosomal secretion. Small GTPases.

[187] Yang Y., Hentati A., Deng H.X., Dabbagh O., Sasaki T., Hirano M., Hung W.Y., Ouahchi K., Yan J., Azim A.C. (2001). The gene encoding alsin, a protein with three guanine-nucleotide exchange factor domains, is mutated in a form of recessive amyotrophic lateral sclerosis. Nat. Genet..

[188] Sellier C., Campanari M.-L., Julie Corbier C., Gaucherot A., Kolb-Cheynel I., Oulad-Abdelghani M., Ruffenach F., Page A., Ciura S., Kabashi E. (2016). Loss of C9ORF72 impairs autophagy and synergizes with polyQ Ataxin-2 to induce motor neuron dysfunction and cell death. EMBO J..

[189] Farg M.A., Sundaramoorthy V., Sultana J.M., Yang S., Atkinson R.A.K., Levina V., Halloran M.A., Gleeson P.A., Blair I.P., Soo K.Y. (2014). C9ORF72, implicated in amytrophic lateral sclerosis and frontotemporal dementia, regulates endosomal trafficking. Hum. Mol. Genet..

[190] Sato T., Iwano T., Kunii M., Matsuda S., Mizuguchi R., Jung Y., Hagiwara H., Yoshihara Y., Yuzaki M., Harada R. (2014). Rab8a and Rab8b are essential for several apical transport pathways but insufficient for ciliogenesis. J. Cell Sci..

[191] Seto S., Sugaya K., Tsujimura K., Nagata T., Horii T., Koide Y. (2013). Rab39a interacts with phosphatidylinositol 3-kinase and negatively regulates autophagy induced by lipopolysaccharide stimulation in macrophages. PLoS ONE.

[192] Pilli M., Arko-Mensah J., Ponpuak M., Roberts E., Master S., Mandell M.A., Dupont N., Ornatowski W., Jiang S., Bradfute S.B. (2012). TBK-1 promotes autophagy-mediated antimicrobial defense by controlling autophagosome maturation. Immunity.

[193] O’Rourke J.G., Bogdanik L., Yáñez A., Lall D., Wolf A.J., Muhammad A.K.M.G., Ho R., Carmona S., Vit J.P., Zarrow J. (2016). C9orf72 is required for proper macrophage and microglial function in mice. Science.

[194] Aoki Y., Manzano R., Lee Y., Dafinca R., Aoki M., Douglas A.G.L., Varela M.A., Sathyaprakash C., Scaber J., Barbagallo P. (2017). C9orf72 and RAB7L1 regulate vesicle trafficking in amyotrophic lateral sclerosis and frontotemporal dementia. Brain.

[195] Hantan D., Yamamoto Y., Sakisaka T. (2014). VAP-B binds to Rab3GAP1 at the ER: Its implication in nuclear envelope formation through the ER-Golgi intermediate compartment. Kobe J. Med. Sci..

[196] Lev S., Ben Halevy D., Peretti D., Dahan N. (2008). The VAP protein family: From cellular functions to motor neuron disease. Trends Cell Biol..

[197] Rocha N., Kuijl C., van der Kant R., Janssen L., Houben D., Janssen H., Zwart W., Neefjes J. (2009). Cholesterol sensor ORP1L contacts the ER protein VAP to control Rab7–RILP–p150Glued and late endosome positioning. J. Cell Biol..

[198] Buratta S., Tancini B., Sagini K., Delo F., Chiaradia E., Urbanelli L., Emiliani C. (2020). Lysosomal Exocytosis, Exosome Release and Secretory Autophagy: The Autophagic- and Endo-Lysosomal Systems Go Extracellular. Int. J. Mol. Sci..

[199] Johnson J.O., Mandrioli J., Benatar M., Abramzon Y., Van Deerlin V.M., Trojanowski J.Q., Gibbs J.R., Brunetti M., Gronka S., Wuu J. (2010). Exome Sequencing Reveals VCP Mutations as a Cause of Familial ALS. Neuron.

[200] Bug M., Meyer H. (2012). Expanding into new markets--VCP/p97 in endocytosis and autophagy. J. Struct. Biol..

[201] Chow C.Y., Landers J.E., Bergren S.K., Sapp P.C., Grant A.E., Jones J.M., Everett L., Lenk G.M., McKenna-Yasek D.M., Weisman L.S. (2009). Deleterious variants of FIG4, a phosphoinositide phosphatase, in patients with ALS. Am. J. Hum. Genet..

[202] Martyn C., Li J. (2013). Fig4 deficiency: A newly emerged lysosomal storage disorder?. Prog. Neurobiol..

[203] Branchu J., Boutry M., Sourd L., Depp M., Leone C., Corriger A., Vallucci M., Esteves T., Matusiak R., Dumont M. (2017). Loss of spatacsin function alters lysosomal lipid clearance leading to upper and lower motor neuron degeneration. Neurobiol. Dis..

[204] Pérez-Brangulí F., Mishra H.K., Prots I., Havlicek S., Kohl Z., Saul D., Rummel C., Dorca-Arevalo J., Regensburger M., Graef D. (2014). Dysfunction of spatacsin leads to axonal pathology in SPG11-linked hereditary spastic paraplegia. Hum. Mol. Genet..

[205] Neumann M., Sampathu D.M., Kwong L.K., Truax A.C., Micsenyi M.C., Chou T.T., Bruce J., Schuck T., Grossman M., Clark C.M. (2006). Ubiquitinated TDP-43 in frontotemporal lobar degeneration and amyotrophic lateral sclerosis. Science.

[206] Gill C., Phelan J.P., Hatzipetros T., Kidd J.D., Tassinari V.R., Levine B., Wang M.Z., Moreno A., Thompson K., Maier M. (2019). SOD1-positive aggregate accumulation in the CNS predicts slower disease progression and increased longevity in a mutant SOD1 mouse model of ALS. Sci. Rep..

[207] Feneberg E., Gray E., Ansorge O., Talbot K., Turner M.R. (2018). Towards a TDP-43-Based Biomarker for ALS and FTLD. Mol. Neurobiol..

[208] Zhang Y.-J., Jansen-West K., Xu Y.-F., Gendron T.F., Bieniek K.F., Lin W.-L., Sasaguri H., Caulfield T., Hubbard J., Daughrity L. (2014). Aggregation-prone c9FTD/ALS poly(GA) RAN-translated proteins cause neurotoxicity by inducing ER stress. Acta Neuropathol..

[209] Picchiarelli G., Dupuis L. (2020). Role of RNA Binding Proteins with prion-like domains in muscle and neuromuscular diseases. Cell Stress.

[210] Hirano A. (1967). Familial Amyotrophic Lateral Sclerosis. Arch. Neurol..

[211] Sun C.N., Araoz C., Lucas G., Morgan P.N., White H.J. (1975). Amyotrophic lateral sclerosis. Inclusion bodies in a case of the classic sporadic form. Ann. Clin. Lab. Sci..

[212] Takahashi K. (1972). Hereditary Amyotrophic Lateral Sclerosis. Arch. Neurol..

[213] Shibata N., Hirano A., Kobayashi M., Siddique T., Deng H.-X., Hung W.-Y., Kato T., Asayama K. (1996). Intense Superoxide Dismutase-1 Immunoreactivity in Intracytoplasmic Hyaline Inclusions of Familial Amyotrophic Lateral Sclerosis with Posterior Column Involvement. J. Neuropathol. Exp. Neurol..

[214] Matsumoto G., Wada K., Okuno M., Kurosawa M., Nukina N. (2011). Serine 403 Phosphorylation of p62/SQSTM1 Regulates Selective Autophagic Clearance of Ubiquitinated Proteins. Mol. Cell.

[215] Ling S.-C., Polymenidou M., Cleveland D.W. (2013). Converging Mechanisms in ALS and FTD: Disrupted RNA and Protein Homeostasis. Neuron.

[216] Mackenzie I.R.A., Bigio E.H., Ince P.G., Geser F., Neumann M., Cairns N.J., Kwong L.K., Forman M.S., Ravits J., Stewart H. (2007). Pathological TDP-43 distinguishes sporadic amyotrophic lateral sclerosis from amyotrophic lateral sclerosis with SOD1 mutations. Ann. Neurol..

[217] Deng H.-X., Chen W., Hong S.-T., Boycott K.M., Gorrie G.H., Siddique N., Yang Y., Fecto F., Shi Y., Zhai H. (2011). Mutations in UBQLN2 cause dominant X-linked juvenile and adult-onset ALS and ALS/dementia. Nature.

[218] Levy J.R., Sumner C.J., Caviston J.P., Tokito M.K., Ranganathan S., Ligon L.A., Wallace K.E., LaMonte B.H., Harmison G.G., Puls I. (2006). A motor neuron disease–associated mutation in p150Glued perturbs dynactin function and induces protein aggregation. J. Cell Biol..

[219] Ayaki T., Ito H., Fukushima H., Inoue T., Kondo T., Ikemoto A., Asano T., Shodai A., Fujita T., Fukui S. (2014). Immunoreactivity of valosin-containing protein in sporadic amyotrophic lateral sclerosis and in a case of its novel mutant. Acta Neuropathol. Commun..

[220] Johnson J.O., Pioro E.P., Boehringer A., Chia R., Feit H., Renton A.E., Pliner H.A., Abramzon Y., Marangi G., Winborn B.J. (2014). Mutations in the Matrin 3 gene cause familial amyotrophic lateral sclerosis. Nat. Neurosci..

[221] Chattopadhyay M., Nwadibia E., Strong C.D., Gralla E.B., Valentine J.S., Whitelegge J.P. (2015). The Disulfide Bond, but Not Zinc or Dimerization, Controls Initiation and Seeded Growth in Amyotrophic Lateral Sclerosis-linked Cu,Zn Superoxide Dismutase (SOD1) Fibrillation. J. Biol. Chem..

[222] Lang L., Kurnik M., Danielsson J., Oliveberg M. (2012). Fibrillation precursor of superoxide dismutase 1 revealed by gradual tuning of the protein-folding equilibrium. Proc. Natl. Acad. Sci. USA.

[223] Chan P.K., Chattopadhyay M., Sharma S., Souda P., Gralla E.B., Borchelt D.R., Whitelegge J.P., Valentine J.S. (2013). Structural similarity of wild-type and ALS-mutant superoxide dismutase-1 fibrils using limited proteolysis and atomic force microscopy. Proc. Natl. Acad. Sci. USA.

[224] Ivanova M.I., Sievers S.A., Guenther E.L., Johnson L.M., Winkler D.D., Galaleldeen A., Sawaya M.R., Hart P.J., Eisenberg D.S. (2014). Aggregation-triggering segments of SOD1 fibril formation support a common pathway for familial and sporadic ALS. Proc. Natl. Acad. Sci. USA.

[225] Abdolvahabi A., Shi Y., Chuprin A., Rasouli S., Shaw B.F. (2016). Stochastic Formation of Fibrillar and Amorphous Superoxide Dismutase Oligomers Linked to Amyotrophic Lateral Sclerosis. ACS Chem. Neurosci..

[226] McAlary L., Aquilina J.A., Yerbury J.J. (2016). Susceptibility of Mutant SOD1 to Form a Destabilized Monomer Predicts Cellular Aggregation and Toxicity but Not In vitro Aggregation Propensity. Front. Neurosci..

[227] Wright G.S.A., Antonyuk S.V., Hasnain S.S. (2016). A faulty interaction between SOD1 and hCCS in neurodegenerative disease. Sci. Rep..

[228] Elam J.S., Taylor A.B., Strange R., Antonyuk S., Doucette P.A., Rodriguez J.A., Hasnain S.S., Hayward L.J., Valentine J.S., Yeates T.O. (2003). Amyloid-like filaments and water-filled nanotubes formed by SOD1 mutant proteins linked to familial ALS. Nat. Struct. Biol..

[229] Ayers J.I., Cashman N.R. (2018). Prion-like mechanisms in amyotrophic lateral sclerosis. Handbook of Clinical Neurology.

[230] Baumer K.M., Koone J.C., Shaw B.F. (2020). Kinetic Variability in Seeded Formation of ALS-Linked SOD1 Fibrils Across Multiple Generations. ACS Chem. Neurosci..

[231] Healy E.F., Roth-Rodriguez A., Toledo S. (2020). A model for gain of function in superoxide dismutase. Biochem. Biophys. Rep..

[232] Vogler T.O., Wheeler J.R., Nguyen E.D., Hughes M.P., Britson K.A., Lester E., Rao B., Betta N.D., Whitney O.N., Ewachiw T.E. (2018). TDP-43 and RNA form amyloid-like myo-granules in regenerating muscle. Nature.

[233] Rhoads S.N., Monahan Z.T., Yee D.S., Shewmaker F.P. (2018). The Role of Post-Translational Modifications on Prion-Like Aggregation and Liquid-Phase Separation of FUS. Int. J. Mol. Sci..

[234] Pokrishevsky E., Grad L.I., Cashman N.R. (2016). TDP-43 or FUS-induced misfolded human wild-type SOD1 can propagate intercellularly in a prion-like fashion. Sci. Rep..

[235] Sproviero D., La Salvia S., Giannini M., Crippa V., Gagliardi S., Bernuzzi S., Diamanti L., Ceroni M., Pansarasa O., Poletti A. (2018). Pathological Proteins Are Transported by Extracellular Vesicles of Sporadic Amyotrophic Lateral Sclerosis Patients. Front. Neurosci..

[236] Nishitoh H., Kadowaki H., Nagai A., Maruyama T., Yokota T., Fukutomi H., Noguchi T., Matsuzawa A., Takeda K., Ichijo H. (2008). ALS-linked mutant SOD1 induces ER stress- and ASK1-dependent motor neuron death by targeting Derlin-1. Genes Dev..

[237] Vance C., Scotter E.L., Nishimura A.L., Troakes C., Mitchell J.C., Kathe C., Urwin H., Manser C., Miller C.C., Hortobágyi T. (2013). ALS mutant FUS disrupts nuclear localization and sequesters wild-type FUS within cytoplasmic stress granules. Hum. Mol. Genet..

[238] Teyssou E., Chartier L., Amador M.-D.-M., Lam R., Lautrette G., Nicol M., Machat S., Da Barroca S., Moigneu C., Mairey M. (2017). Novel UBQLN2 mutations linked to amyotrophic lateral sclerosis and atypical hereditary spastic paraplegia phenotype through defective HSP70-mediated proteolysis. Neurobiol. Aging.

[239] Saeki Y. (2017). Ubiquitin recognition by the proteasome. J. Biochem..

[240] Ryan T.A., Tumbarello D.A. (2018). Optineurin: A Coordinator of Membrane-Associated Cargo Trafficking and Autophagy. Front. Immunol..

[241] Katsuragi Y., Ichimura Y., Komatsu M. (2015). p62/SQSTM1 functions as a signaling hub and an autophagy adaptor. FEBS J..

[242] Chen H.-J., Anagnostou G., Chai A., Withers J., Morris A., Adhikaree J., Pennetta G., de Belleroche J.S. (2010). Characterization of the properties of a novel mutation in VAPB in familial amyotrophic lateral sclerosis. J. Biol. Chem..

[243] Tsai P.C., Liao Y.C., Chen P.L., Guo Y.C., Chen Y.H., Jih K.Y., Lin K.P., Soong B.W., Tsai C.P., Lee Y.C. (2018). Investigating CCNF mutations in a Taiwanese cohort with amyotrophic lateral sclerosis. Neurobiol. Aging.

[244] Türk M., Haaker G., Winter L., Just W., Nickel F.T., Linker R.A., Chevessier F., Schröder R. (2014). *C9ORF72*-ALS: P62- and ubiquitin-aggregation pathology in skeletal muscle. Muscle Nerve.

[245] Shibata N., Hirano A., Kobayashi M., Sasaki S., Kato T., Matsumoto S., Shiozawa Z., Komori T., Ikemoto A., Umahara T. (1994). Cu/Zn superoxide dismutase-like immunoreactivity in Lewy body-like inclusions of sporadic amyotrophic lateral sclerosis. Neurosci. Lett..

[246] Hjerpe R., Bett J.S., Keuss M.J., Solovyova A., McWilliams T.G., Johnson C., Sahu I., Varghese J., Wood N., Wightman M. (2016). UBQLN2 Mediates Autophagy-Independent Protein Aggregate Clearance by the Proteasome. Cell.

[247] Wen X., Tan W., Westergard T., Krishnamurthy K., Markandaiah S.S., Shi Y., Lin S., Shneider N.A., Monaghan J., Pandey U.B. (2014). Antisense proline-arginine RAN dipeptides linked to C9ORF72-ALS/FTD form toxic nuclear aggregates that initiate in vitro and in vivo neuronal death. Neuron.

[248] Kabashi E., Agar J.N., Taylor D.M., Minotti S., Durham H.D. (2004). Focal dysfunction of the proteasome: A pathogenic factor in a mouse model of amyotrophic lateral sclerosis. J. Neurochem..

[249] Williams K.L., Warraich S.T., Yang S., Solski J.A., Fernando R., Rouleau G.A., Nicholson G.A., Blair I.P. (2012). UBQLN2/ubiquilin 2 mutation and pathology in familial amyotrophic lateral sclerosis. Neurobiol. Aging.

[250] Yu L., Chen Y., Tooze S.A. (2018). Autophagy pathway: Cellular and molecular mechanisms. Autophagy.

[251] de Araujo M.E.G., Liebscher G., Hess M.W., Huber L.A. (2020). Lysosomal size matters. Traffic.

[252] Wallroth A., Haucke V. (2018). Phosphoinositide conversion in endocytosis and the endolysosomal system. J. Biol. Chem..

[253] Oakes J.A., Davies M.C., Collins M.O. (2017). TBK1: A new player in ALS linking autophagy and neuroinflammation. Mol. Brain.

[254] Goode A., Butler K., Long J., Cavey J., Scott D., Shaw B., Sollenberger J., Gell C., Johansen T., Oldham N.J. (2016). Defective recognition of LC3B by mutant SQSTM1/p62 implicates impairment of autophagy as a pathogenic mechanism in ALS-FTLD. Autophagy.

[255] Soo K.Y., Sultana J., King A., Atkinson R., Warraich S.T., Sundaramoorthy V., Blair I., Farg M.A., Atkin J.D. (2015). ALS-associated mutant FUS inhibits macroautophagy which is restored by overexpression of Rab1. Cell Death Discov..

[256] Sundaramoorthy V., Walker A.K., Tan V., Fifita J.A., McCann E.P., Williams K.L., Blair I.P., Guillemin G.J., Farg M.A., Atkin J.D. (2015). Defects in optineurin- and myosin VI-mediated cellular trafficking in amyotrophic lateral sclerosis. Hum. Mol. Genet..

[257] Barmada S.J., Serio A., Arjun A., Bilican B., Daub A., Ando D.M., Tsvetkov A., Pleiss M., Li X., Peisach D. (2014). Autophagy induction enhances TDP43 turnover and survival in neuronal ALS models. Nat. Chem. Biol..

[258] Waite A.J., Bäumer D., East S., Neal J., Morris H.R., Ansorge O., Blake D.J. (2014). Reduced C9orf72 protein levels in frontal cortex of amyotrophic lateral sclerosis and frontotemporal degeneration brain with the C9ORF72 hexanucleotide repeat expansion. Neurobiol. Aging.

[259] Mathis S., Goizet C., Soulages A., Vallat J.-M., Le Masson G. (2019). Genetics of amyotrophic lateral sclerosis: A review. J. Neurol. Sci..

[260] Allen S.P., Hall B., Castelli L.M., Francis L., Woof R., Siskos A.P., Kouloura E., Gray E., Thompson A.G., Talbot K. (2019). Astrocyte adenosine deaminase loss increases motor neuron toxicity in amyotrophic lateral sclerosis. Brain.

[261] Boivin M., Pfister V., Gaucherot A., Ruffenach F., Negroni L., Sellier C., Charlet-Berguerand N. (2020). Reduced autophagy upon C9ORF72 loss synergizes with dipeptide repeat protein toxicity in G4C2 repeat expansion disorders. EMBO J..

[262] Stolz A., Wolf D.H. (2010). Endoplasmic reticulum associated protein degradation: A chaperone assisted journey to hell. Biochim. Biophys. Acta Mol. Cell Res..

[263] Atkin J.D., Farg M.A., Walker A.K., McLean C., Tomas D., Horne M.K. (2008). Endoplasmic reticulum stress and induction of the unfolded protein response in human sporadic amyotrophic lateral sclerosis. Neurobiol. Dis..

[264] Vijayalakshmi K., Alladi P.A., Ghosh S., Prasanna V.K., Sagar B.C., Nalini A., Sathyaprabha T.N., Raju T.R. (2011). Evidence of endoplasmic reticular stress in the spinal motor neurons exposed to CSF from sporadic amyotrophic lateral sclerosis patients. Neurobiol. Dis..

[265] Yamanaka T., Nishiyama R., Shimogori T., Nukina N. (2020). Proteomics-Based Approach Identifies Altered ER Domain Properties by ALS-Linked VAPB Mutation. Sci. Rep..

[266] Tran D., Chalhoub A., Schooley A., Zhang W., Ngsee J.K. (2012). A mutation in VAPB that causes amyotrophic lateral sclerosis also causes a nuclear envelope defect. J. Cell Sci..

[267] Cadoni M.P.L., Biggio M.L., Arru G., Secchi G., Orrù N., Clemente M.G., Sechi G., Yamoah A., Tripathi P., Orrù S. (2020). VAPB ER-Aggregates, A Possible New Biomarker in ALS Pathology. Cells.

[268] Schwartz J.C., Ebmeier C.C., Podell E.R., Heimiller J., Taatjes D.J., Cech T.R. (2012). FUS binds the CTD of RNA polymerase II and regulates its phosphorylation at Ser2. Genes Dev..

[269] Buratti E., Baralle F.E. (2001). Characterization and functional implications of the RNA binding properties of nuclear factor TDP-43, a novel splicing regulator of CFTR exon 9. J. Biol. Chem..

[270] Colombrita C., Onesto E., Megiorni F., Pizzuti A., Baralle F.E., Buratti E., Silani V., Ratti A. (2012). TDP-43 and FUS RNA-binding proteins bind distinct sets of cytoplasmic messenger RNAs and differently regulate their post-transcriptional fate in motoneuron-like cells. J. Biol. Chem..

[271] Godena V.K., Romano G., Romano M., Appocher C., Klima R., Buratti E., Baralle F.E., Feiguin F. (2011). TDP-43 Regulates Drosophila Neuromuscular Junctions Growth by Modulating Futsch/MAP1B Levels and Synaptic Microtubules Organization. PLoS ONE.

[272] Herzog J.J., Deshpande M., Shapiro L., Rodal A.A., Paradis S. (2017). TDP-43 misexpression causes defects in dendritic growth. Sci. Rep..

[273] Feiguin F., Godena V.K., Romano G., D’Ambrogio A., Klima R., Baralle F.E. (2009). Depletion of TDP-43 affects *Drosophila motoneurons* terminal synapsis and locomotive behavior. FEBS Lett..

[274] Reber S., Stettler J., Filosa G., Colombo M., Jutzi D., Lenzken S.C., Schweingruber C., Bruggmann R., Bachi A., Barabino S.M. (2016). Minor intron splicing is regulated by FUS and affected by ALS-associated FUS mutants. EMBO J..

[275] Mori K., Lammich S., Mackenzie I.R.A., Forné I., Zilow S., Kretzschmar H., Edbauer D., Janssens J., Kleinberger G., Cruts M. (2013). hnRNP A3 binds to GGGGCC repeats and is a constituent of p62-positive/TDP43-negative inclusions in the hippocampus of patients with C9orf72 mutations. Acta Neuropathol..

[276] Khosravi B., Hartmann H., May S., Mö hl C., Ederle H., Michaelsen M., Schludi M.H., Dormann D., Edbauer D. (2017). Cytoplasmic poly-GA aggregates impair nuclear import of TDP-43 in C9orf72 ALS/FTLD. Hum. Mol. Genet..

[277] Chen Y.P., Huang R., Yang Y., Chen K., Song W., Pan P.L., Li J.P., Shang H.F. (2011). Ataxin-2 intermediate-length polyglutamine: A possible risk factor for Chinese patients with amyotrophic lateral sclerosis. Neurobiol. Aging.

[278] Jutzi D., Akinyi M.V., Mechtersheimer J., Frilander M.J., Ruepp M.-D. (2018). The emerging role of minor intron splicing in neurological disorders. Cell Stress.

[279] Scotti M.M., Swanson M.S. (2016). RNA mis-splicing in disease. Nat. Rev. Genet..

[280] Leblond C.S., Gan-Or Z., Spiegelman D., Laurent S.B., Szuto A., Hodgkinson A., Dionne-Laporte A., Provencher P., de Carvalho M., Orrù S. (2016). Replication study of MATR3 in familial and sporadic amyotrophic lateral sclerosis. Neurobiol. Aging.

[281] Salton M., Elkon R., Borodina T., Davydov A., Yaspo M.-L., Halperin E., Shiloh Y. (2011). Matrin 3 binds and stabilizes mRNA. PLoS ONE.

[282] Yang C., Wang H., Qiao T., Yang B., Aliaga L., Qiu L., Tan W., Salameh J., McKenna-Yasek D.M., Smith T. (2014). Partial loss of TDP-43 function causes phenotypes of amyotrophic lateral sclerosis. Proc. Natl. Acad. Sci. USA.

[283] Kamelgarn M., Chen J., Kuang L., Arenas A., Zhai J., Zhu H., Gal J. (2016). Proteomic analysis of FUS interacting proteins provides insights into FUS function and its role in ALS. Biochim. Biophys. Acta Mol. Basis Dis..

[284] Yu Y., Chi B., Xia W., Gangopadhyay J., Yamazaki T., Winkelbauer-Hurt M.E., Yin S., Eliasse Y., Adams E., Shaw C.E. (2015). U1 snRNP is mislocalized in ALS patient fibroblasts bearing NLS mutations in FUS and is required for motor neuron outgrowth in zebrafish. Nucleic Acids Res..

[285] Rogelj B., Easton L.E., Bogu G.K., Stanton L.W., Rot G., Curk T., Zupan B., Sugimoto Y., Modic M., Haberman N. (2012). Widespread binding of FUS along nascent RNA regulates alternative splicing in the brain. Sci. Rep..

[286] Orozco D., Tahirovic S., Rentzsch K., Schwenk B.M., Haass C., Edbauer D. (2012). Loss of fused in sarcoma (FUS) promotes pathological Tau splicing. EMBO Rep..

[287] Moloney E.B., de Winter F., Verhaagen J. (2014). ALS as a distal axonopathy: Molecular mechanisms affecting neuromuscular junction stability in the presymptomatic stages of the disease. Front. Neurosci..

[288] Boehringer A., Garcia-Mansfield K., Singh G., Bakkar N., Pirrotte P., Bowser R. (2017). ALS Associated Mutations in Matrin 3 Alter Protein-Protein Interactions and Impede mRNA Nuclear Export. Sci. Rep..

[289] Coelho M.B., Attig J., Bellora N., König J., Hallegger M., Kayikci M., Eyras E., Ule J., Smith C.W.J. (2015). Nuclear matrix protein Matrin3 regulates alternative splicing and forms overlapping regulatory networks with PTB. EMBO J..

[290] Zhang X., Yamashita S., Hara K., Doki T., Tawara N., Ikeda T., Misumi Y., Zhang Z., Matsuo Y., Nagai M. (2019). A mutant MATR3 mouse model to explain multisystem proteinopathy. J. Pathol..

[291] Simpson C.L., Lemmens R., Miskiewicz K., Broom W.J., Hansen V.K., van Vught P.W.J., Landers J.E., Sapp P., Van Den Bosch L., Knight J. (2009). Variants of the elongator protein 3 ( ELP3 ) gene are associated with motor neuron degeneration. Hum. Mol. Genet..

[292] Bento-Abreu A., Jager G., Swinnen B., Rué L., Hendrickx S., Jones A., Staats K.A., Taes I., Eykens C., Nonneman A. (2018). Elongator subunit 3 (ELP3) modifies ALS through tRNA modification. Hum. Mol. Genet..

[293] Greenway M.J., Andersen P.M., Russ C., Ennis S., Cashman S., Donaghy C., Patterson V., Swingler R., Kieran D., Prehn J. (2006). ANG mutations segregate with familial and ‘sporadic’ amyotrophic lateral sclerosis. Nat. Genet..

[294] Wu D., Yu W., Kishikawa H., Folkerth R.D., Iafrate A.J., Shen Y., Xin W., Sims K., Hu G. (2007). Angiogenin loss-of-function mutations in amyotrophic lateral sclerosis. Ann. Neurol..

[295] Kirby J., Highley J.R., Cox L., Goodall E.F., Hewitt C., Hartley J.A., Hollinger H.C., Fox M., Ince P.G., Mcdermott C.J. (2013). Lack of unique neuropathology in amyotrophic lateral sclerosis associated with p.K54E angiogenin (ANG) mutation. Neuropathol. Appl. Neurobiol..

[296] Aladesuyi Arogundade O., Stauffer J.E., Saberi S., Diaz-Garcia S., Malik S., Basilim H., Rodriguez M.J., Ohkubo T., Ravits J. (2019). Antisense RNA foci are associated with nucleoli and TDP-43 mislocalization in C9orf72-ALS/FTD: A quantitative study. Acta Neuropathol..

[297] Mehta A.R., Selvaraj B.T., Barton S.K., McDade K., Abrahams S., Chandran S., Smith C., Gregory J.M. (2020). Improved detection of RNA foci in C9orf72 amyotrophic lateral sclerosis post-mortem tissue using BaseScope^TM^ shows a lack of association with cognitive dysfunction. Brain Commun..

[298] Figueroa-Romero C., Hur J., Lunn J.S., Paez-Colasante X., Bender D.E., Yung R., Sakowski S.A., Feldman E.L. (2016). Expression of microRNAs in human post-mortem amyotrophic lateral sclerosis spinal cords provides insight into disease mechanisms. Mol. Cell. Neurosci..

[299] Porta S., Kwong L.K., Trojanowski J.Q., Lee V.M.-Y. (2015). Drosha Inclusions Are New Components of Dipeptide-Repeat Protein Aggregates in FTLD-TDP and ALS *C9orf72* Expansion Cases. J. Neuropathol. Exp. Neurol..

[300] Sobue G., Mcalary L., Taylor J.P., Purice M.D. (2018). Linking hnRNP Function to ALS and FTD Pathology. Front. Neurosci..

[301] Kawahara Y., Mieda-Sato A. (2012). TDP-43 promotes microRNA biogenesis as a component of the Drosha and Dicer complexes. Proc. Natl. Acad. Sci. USA.

[302] Morlando M., Dini Modigliani S., Torrelli G., Rosa A., Di Carlo V., Caffarelli E., Bozzoni I. (2012). FUS stimulates microRNA biogenesis by facilitating co-transcriptional Drosha recruitment. EMBO J..

[303] Waller R., Wyles M., Heath P.R., Kazoka M., Wollff H., Shaw P.J., Kirby J. (2017). Small RNA Sequencing of Sporadic Amyotrophic Lateral Sclerosis Cerebrospinal Fluid Reveals Differentially Expressed miRNAs Related to Neural and Glial Activity. Front. Neurosci..

[304] Campos-Melo D., Droppelmann C.A., He Z., Volkening K., Strong M.J. (2013). Altered microRNA expression profile in amyotrophic lateral sclerosis: A role in the regulation of NFL mRNA levels. Mol. Brain.

[305] Salinas J., Lin H., Aparico H.J., Huan T., Liu C., Rong J., Beiser A., Himali J.J., Freedman J.E., Larson M.G. (2019). Whole blood microRNA expression associated with stroke: Results from the Framingham Heart Study. PLoS ONE.

[306] Boissart C., Nissan X., Giraud-Triboult K., Peschanski M., Benchoua A. (2012). miR-125 potentiates early neural specification of human embryonic stem cells. Development.

[307] Jauhari A., Singh T., Pandey A., Singh P., Singh N., Srivastava A.K., Pant A.B., Parmar D., Yadav S. (2017). Differentiation Induces Dramatic Changes in miRNA Profile, Where Loss of Dicer Diverts Differentiating SH-SY5Y Cells Toward Senescence. Mol. Neurobiol..

[308] Ranjith Sama R.K., Ward C.L., Kaushansky L.J., Lemay N., Ishigaki S., Urano F., Bosco D.A. (2013). FUS/TLS Assembles Into Stress Granules and Is a Prosurvival Factor During Hyperosmolar Stress. J. Cell. Physiol..

[309] Guil S., Cáceres J.F. (2007). The multifunctional RNA-binding protein hnRNP A1 is required for processing of miR-18a. Nat. Struct. Mol. Biol..

[310] Zhang K., Daigle J.G., Cunningham K.M., Coyne A.N., Ruan K., Grima J.C., Bowen K.E., Wadhwa H., Yang P., Rigo F. (2018). Stress Granule Assembly Disrupts Nucleocytoplasmic Transport. Cell.

[311] Kim H.J., Kim N.C., Wang Y.-D., Scarborough E.A., Moore J., Diaz Z., MacLea K.S., Freibaum B., Li S., Molliex A. (2013). Mutations in prion-like domains in hnRNPA2B1 and hnRNPA1 cause multisystem proteinopathy and ALS. Nature.

[312] Buchan J.R., Kolaitis R.-M., Taylor J.P., Parker R. (2013). Eukaryotic stress granules are cleared by autophagy and Cdc48/VCP function. Cell.

[313] Uversky V.N. (2017). The roles of intrinsic disorder-based liquid-liquid phase transitions in the “Dr. Jekyll–Mr. Hyde” behavior of proteins involved in amyotrophic lateral sclerosis and frontotemporal lobar degeneration. Autophagy.

[314] Vanneste J., Vercruysse T., Boeynaems S., Sicart A., Van Damme P., Daelemans D., Van Den Bosch L. (2019). C9orf72-generated poly-GR and poly-PR do not directly interfere with nucleocytoplasmic transport. Sci. Rep..

[315] Brenner D., Weishaupt J.H. (2019). Update on amyotrophic lateral sclerosis genetics. Curr. Opin. Neurol..

[316] Marchetto M.C.N., Muotri A.R., Mu Y., Smith A.M., Cezar G.G., Gage F.H. (2008). Non-Cell-Autonomous Effect of Human SOD1G37R Astrocytes on Motor Neurons Derived from Human Embryonic Stem Cells. Cell Stem Cell.

[317] Haidet-Phillips A.M., Hester M.E., Miranda C.J., Meyer K., Braun L., Frakes A., Song S., Likhite S., Murtha M.J., Foust K.D. (2011). Astrocytes from familial and sporadic ALS patients are toxic to motor neurons. Nat. Biotechnol..

[318] Hooten K.G., Beers D.R., Zhao W., Appel S.H. (2015). Protective and Toxic Neuroinflammation in Amyotrophic Lateral Sclerosis. Neurotherapeutics.

[319] Brites D., Vaz A.R. (2014). Microglia centered pathogenesis in ALS: Insights in cell interconnectivity. Front. Cell. Neurosci..

[320] Mougeot J.-L.C., Li Z., Price A.E., Wright F.A., Brooks B.R. (2011). Microarray analysis of peripheral blood lymphocytes from ALS patients and the SAFE detection of the KEGG ALS pathway. BMC Med. Genom..

[321] Pradat P.-F., Barani A., Wanschitz J., Dubourg O., Lombès A., Bigot A., Mouly V., Bruneteau G., Salachas F., Lenglet T. (2011). Abnormalities of satellite cells function in amyotrophic lateral sclerosis. Amyotroph. Lateral Scler..

[322] Scaricamazza S., Salvatori I., Giacovazzo G., Loeffler J.P., Renè F., Rosina M., Quessada C., Proietti D., Heil C., Rossi S. (2020). Skeletal-Muscle Metabolic Reprogramming in ALS-SOD1G93A Mice Predates Disease Onset and Is A Promising Therapeutic Target. iScience.

[323] Loeffler J.P., Picchiarelli G., Dupuis L., Gonzalez De Aguilar J.L. (2016). The role of skeletal muscle in amyotrophic lateral sclerosis. Brain Pathol..

[324] Morgan S., Duguez S., Duddy W. (2018). Personalized Medicine and Molecular Interaction Networks in Amyotrophic Lateral Sclerosis (ALS): Current Knowledge. J. Pers. Med..

